# Cryo-EM structure of bacteriophage Nocturne116: insights into the architecture of c2-like *Lactococcus* viruses

**DOI:** 10.1128/jvi.00795-26

**Published:** 2026-08-31

**Authors:** Jānis Rūmnieks, Andris Dišlers, Kaspars Tārs

**Affiliations:** 1 National Institute of Research and Innovation, Riga, Latvia; 2 Faculty of Medicine and Life Sciences, University of Latvia 61769, Riga, Latvia; Michigan State University, East Lansing, Michigan, USA

**Keywords:** *Ceduovirus*, *Lactococcus*, cryo-EM, bacteriophage

## Abstract

Nocturne116 is a small bacterial virus with a prolate head and a flexible, non-contractile tail that infects an insect-associated strain of *Lactococcus lactis*. Nocturne116 has little sequence similarity to other bacteriophages and represents a diverged virus lineage that includes the *Lactococcus* phage Q54 and phages of the *Ceduovirus* genus. To better understand the molecular architecture of this group of viruses, we determined the three-dimensional structure of the Nocturne116 virion. The head of the Nocturne116 phage is composed of two types of capsid proteins: the major capsid protein, which assembles into 50 hexamers, and a dedicated vertex protein, which forms 11 pentamers. The particle is stabilized by covalent isopeptide bonds, which result in topologically interlocked capsomer rings in the cylindrical midsection, while in the hemispherical caps, the chainmail-like structure is incomplete. An end vertex of the capsid is occupied by a portal complex, which is connected to the tail via a series of three neck protein rings. The genomic DNA extends through the neck channel up to the second tail tube ring, where it encounters the tail tape measure protein. The tail shaft is built of 26 hexameric rings of the tail tube protein and is capped by an assembly of three minor structural proteins that form a dome-shaped structure at the tail tip. The tail tip lacks characteristic receptor-binding proteins and represents the simplest host-attachment device of any studied *Lactococcus* phage, suggestive of an unusual mechanism by which the virus recognizes and infects its bacterial host.

**IMPORTANCE:**

Viruses that infect the widely used food fermenter *Lactococcus lactis* can cause major problems in the food industry, but despite considerable effort, there are still gaps in the understanding of these viruses. Knowledge of their three-dimensional structure is important for understanding aspects such as particle-stabilizing mechanisms that they employ, or the host recognition mechanisms that these viruses use. The three-dimensional structure of *Lactococcus* phage Nocturne116 reveals a number of unusual features such as two types of capsid proteins making up the head, covalently crosslinked capsomers which, to our knowledge, are the first such observation in a prolate-shaped virus, or a minimalist tail tip which starkly contrasts with the elaborate machinery of other previously studied *Lactococcus* phages. Our results also shed light on the general structure of related ceduoviruses which, despite being the third-largest lactococcal virus group, have remained poorly understood from a structural perspective.

## INTRODUCTION

Lactococci are gram-positive bacteria renowned for their homofermentative ability to produce lactic acid from various sugars, with *Lactococcus lactis*, the most widely known species of the genus, being extensively used for fermentation of milk for production of cheese, sour cream, and other dairy products. Lactococci are widespread in nature and, besides milk or fermented foods, have been isolated from plants, insects, fish, mammals, and wastewater (1). Bacterial viruses—bacteriophages or phages—are equally ubiquitous and are generally found in all environments where bacteria are thriving. For the food industry, phages targeting *Lactococcus* pose a major concern as phage outbreaks can cause large-scale fermentation failures and considerable economic losses (2). Mainly due to these reasons, *Lactococcus* viruses, particularly those infecting industrial strains of *L. lactis*, have been an area of sustained interest, resulting in hundreds of documented phage isolates and considerable effort directed toward understanding the diversity of these viruses, host–phage interactions, and various lactococcal anti-phage defense mechanisms (3).

Of the several evolutionarily distinctive groups of bacteriophages, by far the largest and most prevalent are the tailed viruses (class *Caudoviricetes*). These viruses have a unique and complex architecture of a head (capsid) that encapsulates a double-stranded DNA genome and a tail which mediates adsorption of the virus to its host and delivery of the genome into the cell. The heads of these viruses are commonly isometric with an underlying icosahedral symmetry and can range from about 45 to over 180 nm in diameter to accommodate genome sizes from less than 20 to more than 500 kbp (4, 5); in addition, some bacteriophages have evolved prolate capsids as an adaptation for packaging larger genomes. The tails are even more diverse and, based on their structure, the phages fall into three morphological types with short, noncontractile (podoviruses), long, noncontractile (siphoviruses), or long and contractile (myoviruses) tails (6). All of the isolated *Lactococcus* phages have turned out to be tailed and almost exclusively of the siphovirus morphotype (7). Earlier efforts of categorizing *L. lactis* phages led to recognition of 10 groups of these viruses, of which three, designated “936,” “P335,” and “c2” after representative isolates, collectively accounted for 98% of the known phages, whereas the remaining were termed “rare” (8). Virion structures of phage p2 from the “936” group (now recognized as genus *Skunavirus*), the P335-like phage TP901-1 (currently unclassified), and the “rare” phage 1358 (now *Whiteheadvirus*) have been previously explored using electron microscopy (9–11), although atomic details of their structural proteins were not resolved due to the limited resolution achievable at the time. Additional high-resolution crystallographic studies have been undertaken to investigate the tail tip structures of several *Lactococcus* phages (12–14), which have demonstrated that these viruses often employ unusually large and elaborate baseplate-like assemblies with certain features resembling myoviruses, which contrasts them with the less complex structures commonly observed in gram-negative siphophages (15). No structures of phages from the third-largest “c2” group, now classified in the *Ceduovirus* genus, have been determined, but these viruses appear to have a simpler organization of the tail tip without an observable baseplate-like structure.

During our previous efforts aimed at exploring the diversity of insect-associated bacteria and their phages, we isolated a strain of *Lactococcus lactis* and a corresponding bacteriophage Nocturne116 (16). Nocturne116 is a small phage with a prolate, 60-by-40 nm head and a flexible, ~110 nm long tail, consistent with a typical siphovirus morphology. The genome sequence of the phage shows little similarity to other viruses except bacteriophage Q54 (17), the sole representative of one of the uncommon *Lactococcus* phage groups now recognized as the *Questintvirus* genus; however, Nocturne116 does not reach the similarity threshold to be included in that genus. Morphologically, Nocturne116 and Q54 virions appear virtually identical, and they closely resemble those of ceduoviruses (18) and the *Lactococcus garvieae* phage GE1 (19) from the *Chersteyvirus* genus, which have similarly sized prolate heads but slightly differ in the length of their tails. In addition, these viruses have comparably sized genomes with similarities in genome organization, suggesting that Nocturne116, Q54, GE1, and the ceduoviruses are all representatives of a single, highly diverged lactococcal virus lineage.

Here, we have used cryogenic electron microscopy (cryo-EM) to determine the high-resolution structure of the Nocturne116 virion. The structure documents in detail the molecular architecture of an unusual *Lactococcus* virus and provides insight into the broader organization of the c2-like viruses.

## RESULTS

### Overall structure

**Fig 1:**
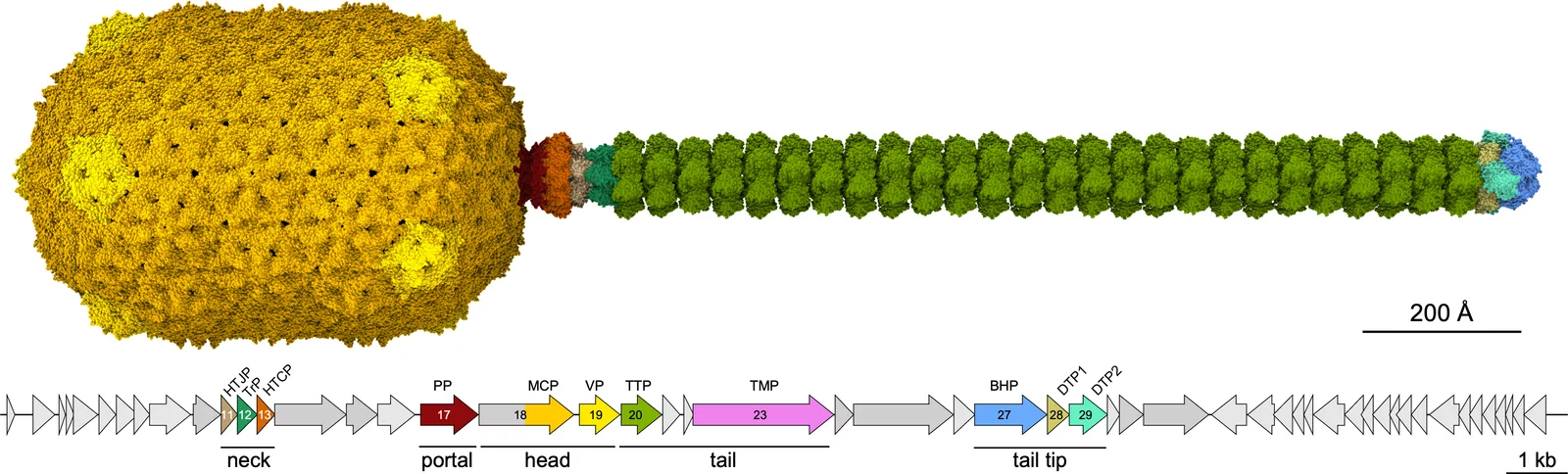
Overall structure of the Nocturne116 bacteriophage. A composite model of the virion, constructed by fitting together refined molecular models of the viral subcomponents and a symmetry-generated model of the tail. Below, the genome structure of the Nocturne116 bacteriophage, with open reading frames (ORFs) encoding the structural proteins indicated in color. ORF numbers and protein abbreviations used in the text are indicated inside and above the arrows, respectively.

The structure of the Nocturne116 bacteriophage was determined as a combination of focused cryo-EM reconstructions of the head, head-to-tail interface (neck), tail tube, and the tail tip, taking advantage of the smaller size and respective symmetry of each subcomponent. Except for the packaged genome and contents of the tail lumen, all components of the bacteriophage could be resolved at a sufficiently high resolution (2.63–3.53 Å) to allow building of an atomic model of the entire virion (Fig. 1). In total, the particle contains eleven types of identifiable structural proteins of which two form the head, four are involved in the head-to-tail interface, and the remaining five make up the tail of the phage.

### Head

**Fig 2:**
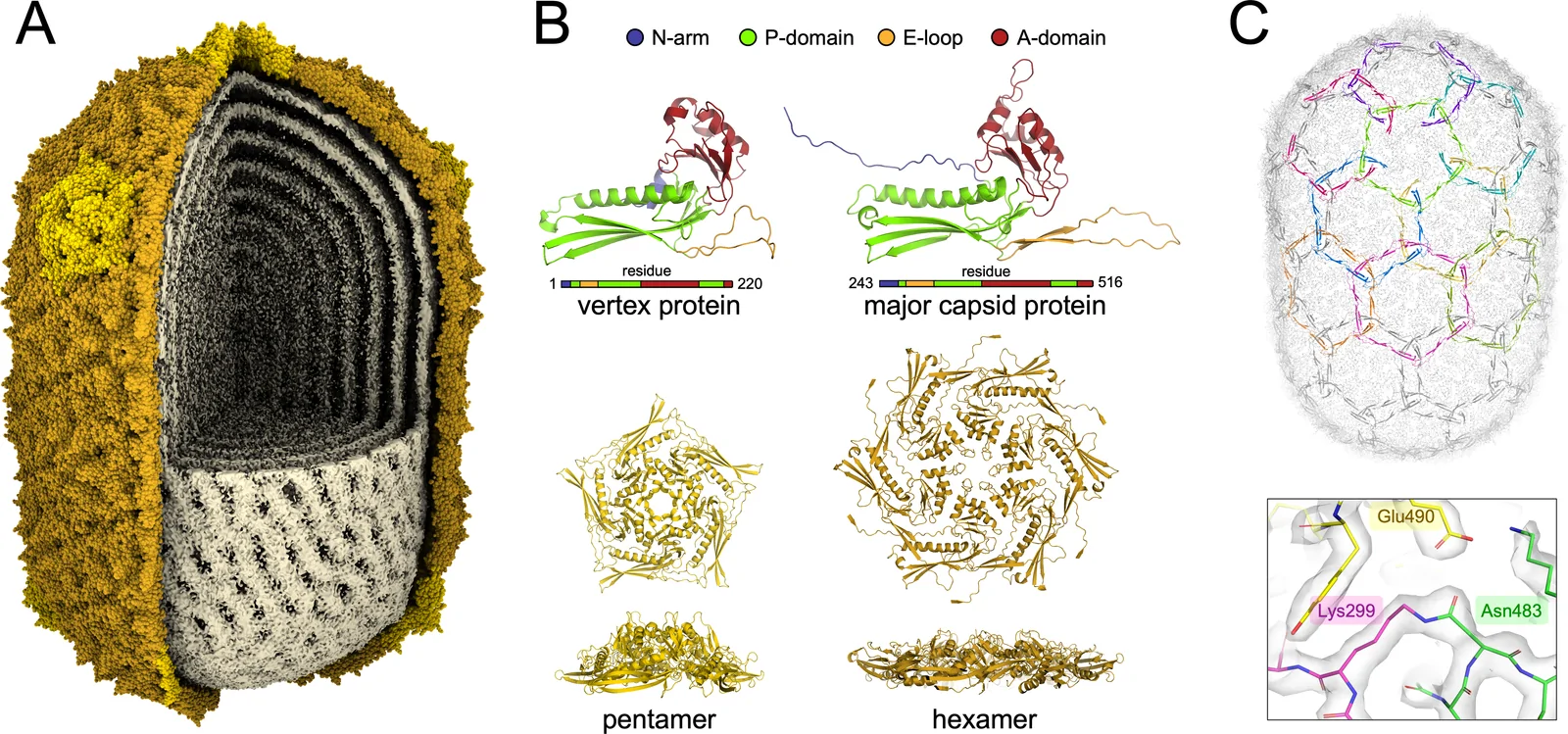
Structure of the Nocturne116 head. (**A**) Composite model of the head. The capsid is rendered as a sphere model with the MCP colored gold and the VP yellow. Part of the model is removed to reveal the internal DNA organization as observed in the fivefold-averaged reconstruction of the head. (**B**) Structure of the capsid proteins. The domain organization of the two protein species is indicated. The bottom panel shows structure of the pentameric VP and hexameric MCP capsomers in the particle. (**C**) Covalent crosslinks between MCP and VP subunits in the Nocturne116 particle resulting from isopeptide bonds near the quasi-threefold symmetry axes. The peripheral β-strands of subunit P-domains are highlighted to emphasize the structure of topologically interlocked capsomer rings. The bottom panel provides a close-up view of the isopeptide bond, with the residues involved in formation of the bond highlighted and density around the covalent bond shown.

The prolate head of the Nocturne116 bacteriophage is approximately 650 Å long and 430 Å wide and consists of a ~20 Å thick outer protein shell which holds a tightly packed genome, observed as eight concentric layers beneath the capsid surface (Fig. 2A). The underlying geometry of the protein shell corresponds to an icosahedron elongated along its fivefold axis in which the subunits are arranged according to triangulation numbers *T*_end_ = 4, *T*_mid_ = 8. The capsid is constructed of two types of protein: the major capsid protein (MCP), which assembles into hexamers, and another protein species, which we have designated the vertex protein (VP), which exclusively forms pentamers at the vertices of the particle. In total, the capsid contains 355 subunits: 50 MCP hexamers and 11 VP pentamers.

In the Nocturne116 genome, the MCP is encoded as the C-terminal domain of the ORF18 protein, which additionally contains an N-terminal prohead protease and a putative central scaffolding domain (Fig. S1A). In the mature head, the observed N-terminus of the MCP corresponds to residue 243 of the ORF18 protein, suggesting that the protease cleaves the precursor between Arg242 and Ser243 at the junction of the scaffolding and MCP domains. The overall structure of the MCP closely follows the canonical HK97 fold found in all tailed phage MCPs (20) and consists of an extended N-terminal arm, peripheral (P) and axial (A) domains, and a distinctive extended (E) loop emanating from the P-domain (Fig. 2B). Irrespective of their location in the particle, the conformation of MCP monomers is similar, and notable differences are only observed in the positioning of the E-loops and N-arms (Fig. S2A). Depending on the local capsid curvature, the E-loops are bent at a different angle relative to the rest of the protein (Fig. S2B), while the N-arms adopt either of two general conformations: in most MCP subunits, the nine N-terminal residues extend away from the protein and make contact with an E-loop from a neighboring hexamer, while in MCPs adjacent to VP pentamers or to the portal, the N-arm is bent back at an approximately 60° angle and makes a similar interaction with an MCP in the same hexamer.

The VP is homologous to the MCP, although both proteins share only about 24% sequence identity (Fig. S1B). Likewise, the VP adopts the HK97 fold, and despite the diverged sequences, the core structure of the two proteins is very similar (Fig. 2B). The VP is the product of the adjacent ORF19 which, in contrast to ORF18, does not encode additional protease or scaffolding domains. With a length of 220 residues, the VP is 54 residues shorter than the MCP domain of ORF18, with the main savings coming from a significantly smaller E-loop, a shortened loop at the tip of the A-domain and, notably, almost complete elimination of the N-arm. The higher-order structure of the two proteins, however, is more distinctive: while the MCP hexamers are relatively thin and flat, VP subunits in the pentamers lie at a considerably steeper angle, which manifests in ~15 Å protrusions at the vertices of the capsid (Fig. 2B).

Within both types of capsomers (hexamers and pentamers), contacts between adjacent subunits are formed between the A-domains and by the E-loops, which extend over P-domains of neighboring monomers, while interactions between capsomers involve various regions of the P-domains and, in hexamers, also the N-terminal arms. Notably, subunits from nearby capsomers are additionally linked via covalent isopeptide bonds between a lysine residue in the E-loop and an asparagine side chain at the distal end of the P-domain. The covalent bonds are formed around the local quasi-threefold axes where three hexamers meet, between Lys299 of one MCP monomer and Asn483 of another, while a nearby glutamate residue (Glu490) implicated in the catalytic mechanism of the bond formation (21) is contributed by the third subunit (Fig. 2C). The VP has an equivalent asparagine residue (Asn196) in the P-domain which forms an isopeptide bond with Lys299 from an adjacent MCP; however, the E-loop of the VP does not contain any lysine residues and, due to its smaller size, is unable to reach the nearby quasi-threefold axis. Each VP molecule is therefore covalently bonded only to a single MCP and consequently, while the MCP subunits in the cylindrical midsection are topologically arranged as catenated rings, the “chainmail” in the caps is incomplete due to the missing VP-MCP crosslinks (Fig. 2C). Consistent with the structural data, denaturing gel electrophoresis of Nocturne116 virions reveals no monomeric MCP species; instead, the protein is observed in various oligomeric states (Fig. S3A). Aberrant electrophoretic mobility of the non-linear, crosslinked molecules makes assignment of particular oligomeric species to each of the gel bands unreliable, but we note that the dominant 90 and 180 kDa gel bands, in which a tryptic peptide corresponding to the VP was detected by mass spectrometry, are consistent with a VP linked to a chain of two or five MCP subunits (predicted molecular weight: 84 and 174 kDa, respectively) which are the major covalently linked oligomeric species observed in the capsid structure (Fig. S3B and C).

### Portal

**Fig 3:**
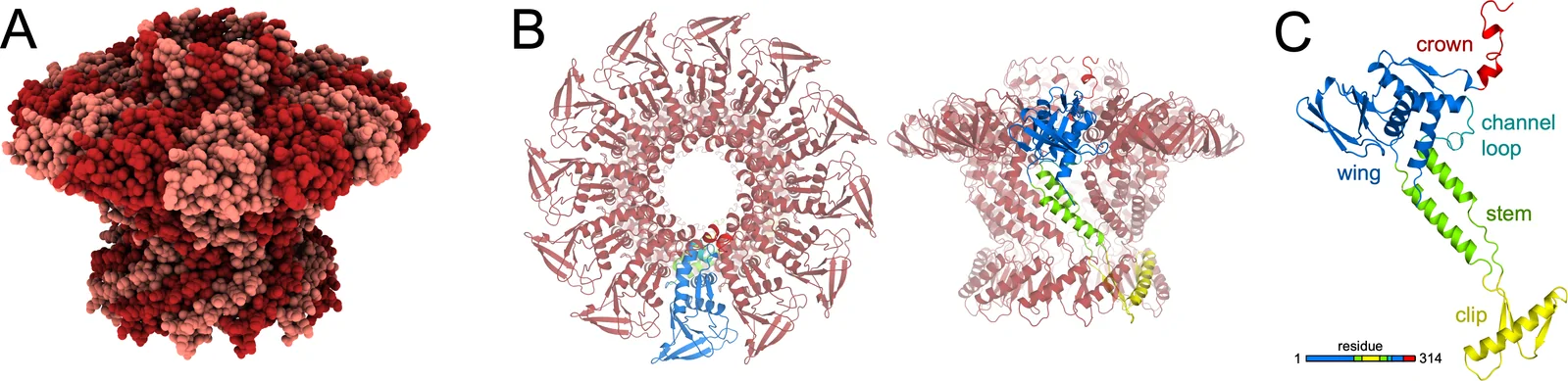
Structure of the Nocturne116 portal. (**A**) Model of the portal assembly with the PP monomers colored in alternating shades of red. (**B**) Top and side views of the portal with one of the monomers colored according to the domain structure of the protein. (**C**) Domain structure of the PP.

Instead of a VP pentamer, one of the end vertices of the Nocturne116 capsid contains a portal that provides a gateway for genome entry and release from the head. The portal complex of the Nocturne116 phage has the typical toroidal shape observed in tailed phage particles (22) and is built from 12 molecules of the 314-residue-long portal protein (PP), encoded by ORF17 in the viral genome (Fig. 3A and B). Like other structurally characterized tailed phage PPs, the Nocturne116 protein has recognizable clip, stem, wing, and crown domains (Fig. 3C). The clip domains are positioned at the distal end of the portal where they form small three-stranded β-sheets, covered from outside by an array of slanted 30-Å long α-helices. Two antiparallel α-helices of the stem domain connect the clip with the larger wing region, which is located inside the head and consists of a central α-helical core and a peripheral all-β subdomain. The tunnel loop, a segment between the stem and wing domains implicated in DNA containment in the head (23), is 10 residues long in the Nocturne116 PP, contains a small α-helix, and is folded back toward the inner wall of the portal channel. The 30 C-terminal residues of the PP correspond to the crown domain, of which the 20 wing-proximal residues form an approximately 20-Å tall ring around the channel, while the remaining residues, which extend deeper into the head, are not visible in the reconstruction. Of note, the 34 N-terminal residues of the PP, which are positioned on the particle exterior, are also disordered and not observable in the map.

The portal contacts the capsid through the wing domains, which in the pressurized, DNA-filled head are pushed against the P-domains of the surrounding MCP molecules. Due to the mismatching fivefold and twelvefold symmetries of the two components, the interaction is asymmetric, and contacts between individual PP and MCP subunits at each position are different. To visualize the portal–capsid interaction in the Nocturne116 virion, we performed an asymmetric reconstruction of the portal-containing vertex, which, however, revealed no major conformational changes among the MCP and PP monomers despite the different local environments (Fig. S4). Most of the contacts between the portal and the capsid are formed between the peripheral β-sheets of PP wing domains and various regions of MCP P-domains, but the conformational changes for accommodating the different interactions are almost exclusively done by the PP; the adjustments mainly involve reorientation of some of the bulkier side chains, and only a single PP monomer adopts a slightly different backbone conformation in a peripheral loop (Fig. S4B and C). The other notable portal–capsid interaction takes place between the E-loop-opposite end of the MCP and the base of the wing; at this interface, a loop in the P-domain undergoes a conformational change in two of the five surrounding MCP molecules (Fig. S4E and F).

### Neck

**Fig 4:**
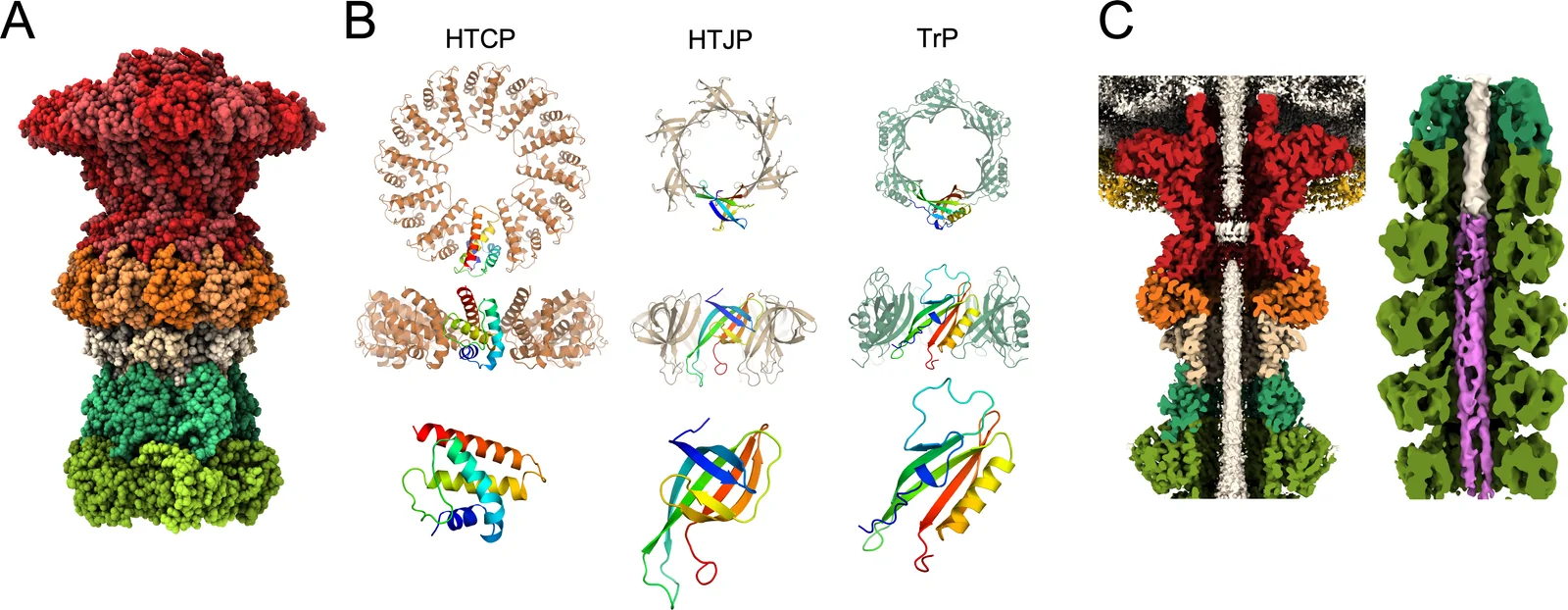
Structure of the Nocturne116 neck. (**A**) Model of the head-to-tail interface. The portal is shown in red; rings of the HTCP, HTJP, and TrP in orange, tan, and sea green, respectively; and the first tail tube ring in olive green. (**B**) Structure of the neck proteins. Neck protein rings are shown as viewed from the top and the side, with one of the subunits rainbow-colored blue (N-terminus) to red (C-terminus). The bottom row presents an enlarged view of the rainbow-colored monomers. (**C**) Density of the genomic DNA and tail tape measure protein (TMP) inside the neck (left) and neck-proximal tail tube (right). Density corresponding to the neck proteins is colored consistently with panel A; the DNA density is shown in off-white and the TMP in light magenta.

The portal is connected to the tail tube via a series of three protein rings (Fig. 4A), each formed by one of the neck proteins, which we have designated the tail terminator protein (TrP), head–tail joining protein (HTJP), and head–tail connector protein (HTCP) by following the nomenclature used for phage 80α (24). The neck proteins are encoded by ORF11, ORF12, and ORF13 in the phage genome which, somewhat atypically, are located amid DNA replication proteins outside of the structural gene operon (Fig. 1). Both the tail tube-proximal TrP and intermediate HTJP rings are built of hexamers of the respective proteins, while the HTCP ring consists of 12 subunits like the portal and serves as an adaptor for connecting the dodecameric portal to the sixfold-symmetrical tail (Fig. 4B). Within the virion, the stacked TrP, HTJP, and HTCP rings form a hollow 85-Å tall structure with a 35-Å wide inner channel; the width of the channel is virtually constant, while the outer diameter of the neck ranges from approximately 55 Å near the TrP-HTJP interface to over 100 Å at the widest point of the HTCP ring.

The TrP and HTJP have weak sequence identity and show some similarities in structure, which suggests that the two proteins are homologous. The TrP is 108 residues long and has a core structure of a five-stranded β-sheet with an α-helix inserted in a loop between two of the β-strands, while the HTJP is shorter (88 residues) and consists of a single six-stranded β-barrel. The TrP and HTJP share a common feature of a four-stranded antiparallel β-sheet that lines the internal channel, while the more dissimilar regions of the two proteins are positioned on the outside of the neck. The HTCP spans 96 residues and is an all-α protein composed of four α-helices, which fold into a wedge-shaped globular domain. The portal-facing side of the HTCP ring is shaped by the C-terminal (α4) helices of the protein, which are radially arranged on top of the ring like spokes of a wheel. Pockets formed between adjacent α4 helices serve as the docking sites for the PP, which binds to the HTCP through a peripheral loop in the clip domain. On the opposite side of the ring, two HTCP molecules cooperatively bind an HTJP monomer, which is achieved by two alternating states of the HTCP, each posed to interact with a different region of the HTJP; the two HTCP configurations differ in positioning of the loop between the two C-terminal (α3 and α4) helices and in the conformation of some of the nearby side chains.

### Genomic terminus

The cryo-EM reconstructions of the head-to-tail interface reveal strong but featureless and rotationally averaged cylindrical density inside the neck (Fig. 4C, left), which we assign to the terminus of the double-stranded DNA genome protruding from the head. The density tapers off and disappears near the clip and stem regions of the portal but reemerges at the narrowest point of the portal channel as a strong, 20 Å-diameter donut-shaped feature, suggestive of an interaction between the portal and the DNA at that location. The DNA density extends further down the tail tube through the first head–proximal tail ring and is then followed by another structure with extended tubular features and a hollow core (Fig. 4C, right), consistent with the expected appearance of the tail tape measure protein (TMP), an elongated, trimeric α-helical protein that extends through the entire tail lumen. However, due to the flexibility of the tail tube, the apparently arbitrary orientation of the DNA and TMP in relation to the symmetrically arranged neck protein subunits, and the limited number of particles, we were unable to resolve further higher-resolution details of the DNA/TMP complex.

### Tail tube

**Fig 5:**
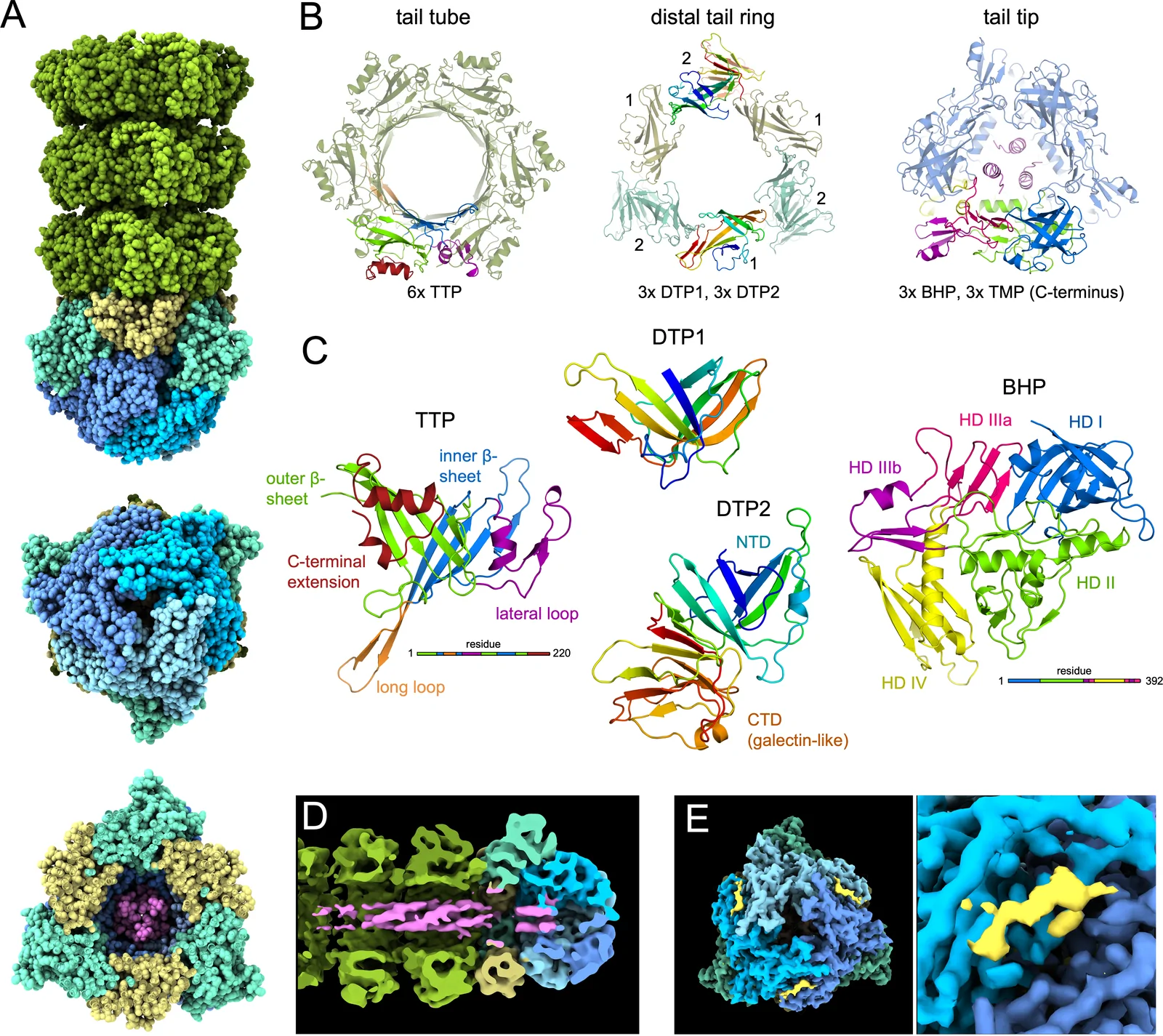
Structure of the Nocturne116 tail. (**A**) Model of the tail tube and tail tip. The TTP is shown in green; the distal tail protein 1 (DTP1) and distal tail protein 2 (DTP2) in khaki and aquamarine, respectively; the baseplate hub protein (BHP) in shades of blue; and the C-terminal helix of the tail tape measure protein in light magenta. The top, middle, and bottom panels show the structure from the front, the bottom, and cut across the distal tail protein ring looking toward the tail tip. (**B**) Structure of the tail protein rings. The TTP, DTP1/DTP2, and BHP assemblies are shown from the top, with one subunit of each colored as in panel C. (**C**) Structure of the tail proteins. The TTP and BHP are colored according to their indicated functional regions, while the distal tail proteins are rainbow-colored blue (N-terminus) to red (C-terminus). (**D**) Density of the TMP inside the distal tail lumen. The tail tip reconstruction is low-pass-filtered to 6 Å for better interpretability. Density attributable to the different structural proteins is colored as in panel A. (**E**) Unaccounted density near the surface of the tail tip. An unsharpened map of the tail tip reconstruction is shown with the density within 3 Å of the fitted model colored as in panel A. Density in BHP pockets not attributable to the protein is shown in yellow. The left panel shows the entire tail tip as viewed from the bottom; the right panel provides a close-up view of one of the pockets.

ORF20 in the Nocturne116 genome encodes a 220-residue tail tube protein (TTP), the major structural component of the tail. The structure of the TTP is consistent with the typical *Caudoviricetes* tail tube protein fold (25), defined by a central β-sandwich, a peripheral α-helix, and a characteristic long loop. The central part of the Nocturne116 TTP is comprised of two four-stranded antiparallel β-sheets, arranged as a β-sandwich, and three flanking α-helices, while the peripheral regions are dominated by several prominent loops, most notably a 20-residue segment between strands β2 and β3 which extends more than 35 Å away from the β-sandwich, a ~20 Å long loop connecting strands β8 and β9, and a 20-residue stretch between α1 and β6 which contains a short β-hairpin structure (Fig. 5C).

The tail tube of the phage consists of 26 TTP rings, stacked at a 42.8 Å distance along the central axis and each rotated by 16.1° with respect to the previous ring. Each ring is formed by six TTP subunits which assemble into a nut-shaped structure with a slightly angular, 35–40 Å wide internal channel and a hexagonal outline with a diameter of 75–110 Å (Fig. 5B). Within the ring, the subunits are positioned with one of the β-sheets facing the tail lumen in a way that strands β2 and β6 from adjacent monomers join and create a continuous 24-stranded β-barrel that lines the interior of the ring (Fig. S5A and B). The other sheet of the β-sandwich has its lateral strand bonded to the β-hairpin in the α1-β6 loop from the neighboring subunit, which results in a six-stranded β-sheet in the tail-assembled state (Fig. S5C). This β-sheet is additionally decorated by the 30-residue C-terminal segment, which contains two short α-helices and is an integral part of the central domain with a shared hydrophobic core. Like in other previously studied bacteriophage TTPs, the long loops between strands β2 and β3 in the Nocturne116 protein serve to connect adjacent rings in the tail. The inter-ring contacts are mediated only via the distal regions of the loops which fit into pockets collectively formed by three monomers in the adjacent ring (Fig. S5D and E), while the stem of the loop is unconstrained and has a considerable freedom of movement, consistent with the observed flexibility of the tail. At lower contour levels, averaged-out density of the TMP is also observable in the lumen of the tail tube (Fig. S5F).

### Tail tip

The distal end of the Nocturne116 tail contains an assembly of three minor tail proteins which form a terminal tail ring and a dome-shaped structure that caps the end of the tube (Fig. 5A). The complex spans 80 Å from the final TTP ring to the end of the tip, has a width of 90 Å near the tail tube and 30 Å at the tip, and features three ~20 Å protrusions in the middle which give the structure a roughly triangular appearance when viewed down the central axis. The inner channel starts with the same width as in the tail tube and gradually becomes narrower toward the tip; however, the tip is not completely sealed but leaves a small triangular opening that connects the tail lumen with the outside environment.

The terminal ring at the end of the tail tube is built of two distal tail proteins (DTPs), which we refer to as DTP1 and DTP2. The ring is composed of three DTP1 and three DTP2 molecules in an alternating order which reduces the sixfold symmetry of the tail tube to threefold (Fig. 5B). The 121-residue DTP1 is encoded by ORF28 in the phage genome and has an all-β architecture with nine strands arranged as a β-sandwich (Fig. 5C). The sandwich has a TTP-like topology but has an additional strand in the outer β-sheet, lacks the peripheral α-helix, and the long loop between strands β2 and β3 is reduced to only seven residues. The DTP2, product of ORF29, consists of two domains (Fig. 5C), of which the 100-residue N-terminal domain (NTD) is integrated into the ring wall while the C-terminal domain (CTD), formed by residues 101–207, is attached to the outer surface of the tail tip. The NTD presents another variation of the TTP fold in which the β2 strand has hydrogen-bonded with β1, and β5 with β7 to form a squashed, six-stranded β-barrel with two additional lateral β-strands. The α-helix between strands β3 and β4 is present but, like in DTP1, the loop between strands β2 and β3 is much shorter and, instead of pointing downwards, forms part of the inner wall of the distal tail ring. The CTD is organized as a β-sandwich, constituted of six- and three-stranded antiparallel β-sheets, and a short flanking α-helix. The topology of the CTD is different from the TTP and instead bears similarity to the galectin-like fold previously observed in some gram-positive phage tail proteins (12, 26). The two DTP2 domains are bound to one another via numerous, predominantly hydrophobic interactions, suggesting that the CTD is not mobile and likely remains affixed to the outer wall of the tail.

The cap of the tail is formed by a trimer of the ORF27 product which, due to its lack of similarity to proteins of known function, was originally annotated as a “minor structural protein” in the viral genome. The determined three-dimensional structure of the protein revealed clear similarities with the baseplate hub protein (BHP), a large multi-domain protein broadly conserved in the tailed bacteriophages. The BHPs share a common organization of four hub domains (HDs) (27), of which HD I and HD III have a highly conserved, β-barrel or β-sandwich-like structure and are positioned below the distal tail ring, whereas the peripheral HD II and HD IV domains adopt a more variable α/β architecture and are involved in closure of the tail tube. The overall organization of the Nocturne116 protein is similar, although there are some notable deviations. The HD I of the Nocturne116 BHP (residues 1–95) folds into a six-stranded β-barrel, with five of the strands lining the inner wall of the tail tip chamber as a twisted β-sheet. The HD II (residues 96–224) contains five short α-helical segments in the central part but is otherwise largely devoid of secondary structure and consists mostly of ordered loop regions. The normally highly conserved HD III is the most diverged in Nocturne116 and has been split into two subdomains, which we have designated HD IIIa and HD IIIb. HD IIIa is collectively formed by three BHP regions (residues 241–256, 347–360, and 376–391) and consists of a three-stranded antiparallel β-sheet and several ordered loops. The HD IIIa is located at the position where HD III is normally found, with the β-sheet of the subdomain contributing three additional strands to the interior-facing sheet from the nearby HD I. HD IIIb (residues 225–240 and 361–375) is slightly smaller and consists of a three-stranded β-sheet and a short α-helix. The polypeptide chain traverses the two subdomains several times, with the underlying topology suggestive of a single ancestral β-sandwich domain which, during the course of evolution, has opened like a clamshell to produce two separate β-sheets. The HD IIIb is positioned on the exterior of the tail tip, with its β-sheet sitting on top of HD IV and the α-helix involved in extensive contacts with the galectin-like domain of the DTP2. The HD IV (residues 257–346) has an α/β architecture with a four-stranded β-sheet and two α-helices. HD IV and HD II collectively form the very tip of the tail, with the two domains arranged in an alternating pattern around the central axis. HD IV forms extensive contacts with HD II from the neighboring subunit but few interactions with that from the same molecule, which manifests in a noticeable groove between the two domains near the tip. Interestingly, an elongated density feature was observed in a pocket near the HD II–HD IV interface (Fig. 5E), the origin of which has currently remained unknown. In contrast to the surrounding BHP density, the feature is weaker and of lower resolution, indicating low occupancy and/or low specificity of the bound substance.

The reconstruction of the tail tip revealed additional density of three α-helices bound to the BHP inside the tail tip chamber and further lower-resolution features akin to a trimeric coiled coil in the nearby portion of the tail tube (Fig. 5D) but not inside the distal tail ring. The BHP-bound high-resolution features were clearly recognized as the C-termini (residues 748–765) of the TMP (Fig. 5A and B), which, according to an *in silico* model, consists entirely of α-helical segments interspersed with flexible linker regions. In the C-terminal region, the terminal TMP helix is predicted to be preceded by a 25-residue linker and another 30-residue α-helix, which is consistent with the observed density features in the distal tail ring and the tail tube, respectively. Further focused reconstructions of the tail tube did not reveal interpretable higher-resolution features, suggesting that the TMP forms only weak, sequence-nonspecific interactions with the TTP.

## DISCUSSION

The three-dimensional structure of the Nocturne116 bacteriophage presents a number of unusual features which together define the distinctive architecture of a “rare” lactococcal virus lineage. Yet, as structural features are often conserved far beyond recognizable sequence identity, the Nocturne116 structure can provide additional insight into the organization of other c2-like viruses and help to better understand the diversity and evolution of *Lactococcus* bacteriophages.

Structurally, the MCP is among the most conservative of the tailed bacteriophage proteins, with the underlying HK97 fold maintained in their distant relatives of archaeal (28) and even eukaryotic (29) dsDNA viruses. It is therefore unsurprising that, despite the diverged sequence, the monomer structure of the Nocturne116 MCP is very similar to capsid proteins of a wide range of bacteriophages infecting both gram-positive and gram-negative bacteria. However, a much more unusual feature of the Nocturne116 phage is the use of a specialized version of the MCP—a dedicated “vertex protein”—for assembling the pentameric capsid protein species in the particle caps. Such two-protein capsid architecture appears to be common in all c2-like phages: the *Lactococcus* phages c2, GE1, and Q54 all encode a hypothetical protein with an unannotated function after their MCP, which, upon examination, has weak sequence similarity with the MCP and a predicted HK97-like fold, suggesting that these likely function as VPs in the other phages as well.

Viruses with icosahedral capsids commonly employ a single type of protein to build particles with “quasi-equivalent” symmetry, in which the subunits attain various conformations to adapt to the local geometric environment within the particle (30). Alternatively, a virus might encode separate protein species specialized for different conformational states, which is typical for some virus groups (31, 32) but rarely observed in the tailed bacteriophages. Use of different capsid proteins for building hexamers and pentamers has been previously documented in the large *Escherichia* bacteriophage T4; the two proteins—gp23 and gp24—are homologous and have roughly the same level of sequence identity as the MCP and VP of the Nocturne116 phage (33). Still, the existence of multiple capsid proteins in a small and otherwise structurally simple virus is unusual, and the evolutionary benefit for such a solution is unclear. Compared to isometric capsids, assembly of prolate-shaped particles is more complicated, as not only a specific diameter but also a particular length of the particle needs to be achieved. The capsid proteins are also required to support additional conformational states in the cylindrical midsection; however, this does not appear to be a major limitation as the prolate phage capsids are commonly assembled from a single type of MCP (34–37) like their isometric counterparts. Nevertheless, as the emergence of an additional capsid protein type in the c2-like phages appears to coincide with the appearance of prolate-shaped capsids in this virus group, it is tempting to speculate that the two events might be related. In this respect, it is interesting to note that assembly of the normally prolate capsid of the *Staphylococcus* prophage φ12 can be switched to isometric by a satellite phage element which encodes an alternative version of the MCP (36); this alternate MCP only forms pentamers which can incorporate into the φ12 procapsids and derail the formation of prolate particles. It could be hypothesized that the VPs of the c2-like phages might act in the opposite way by somehow switching assembly to a prolate shape, but experimental evidence is required to test this possibility.

Covalently crosslinked protein subunits are another uncommon feature found in the Nocturne116 particle and, to our knowledge, are the first instance where such crosslinks have been observed in a prolate-shaped capsid. Crosslinks resulting in topologically interlocked protein rings were first observed in the *Escherichia* bacteriophage HK97, which was also the first determined atomic-resolution structure of any tailed phage capsid (38). The HK97 isopeptide bonds are formed by a mechanism in which the amino group of a lysine residue becomes a nucleophile by transferring a proton to a nearby glutamate and then attacks the γ-carbon of an asparagine side chain to form the covalent bond (21). Although the lysine, glutamate, and asparagine residues in the Nocturne116 MCP have probably arisen independently as a result of convergent evolution, their similar location suggests that the underlying catalytic mechanism of the isopeptide bond formation is likely the same. Since HK97, the growing body of bacteriophage structures has uncovered a range of other capsid-stabilizing mechanisms, such as various cement and capsid decoration proteins (39), but the originally observed crosslinked capsid proteins have remained an exception rather than the norm. A more recent high-throughput structural study revealed HK97-like isopeptide bonds in several actinobacterial viruses (40); however, in that case, the covalent linkages were not conserved within a group of closely related viruses, which raised questions about their importance in capsid stability. Besides Nocturne116, covalently linked MCP subunits have been detected both in the c2 phage and in the c2-like GE1 and Q54 viruses (17–19), where, despite the low overall sequence identity among the MCPs, the asparagine, lysine, and glutamate residues involved in the crosslink formation are all universally conserved, suggesting that these crosslinks are much more important and perhaps essential in the c2-like viruses. Sequence similarity among the c2-like VPs is barely detectable, but a peripheral asparagine residue in the P-domain is also universally conserved, suggesting that the MCP-VP crosslinks are present in the other phages as well. In contrast to Nocturne116, a lysine residue is present in the E-loop of the c2 and Q54 VPs; however, the size of the loop is not increased, and the catalytic glutamate in the P-domain is likewise absent in either phage, suggesting that the incomplete chainmail in the caps is another shared feature in this virus lineage. Curiously, in a cylindrical pressure vessel, the side walls experience approximately twice as much stress as the hemispherical caps (41); thus, it might be relevant for the phage to have only this part of the capsid additionally reinforced.

Like the MCP, the portal protein is another highly conserved structural component of the tailed phage particles. The Nocturne116 PP has a rather minimalistic crown domain and shows some divergence in the peripheral crown region, but otherwise is not particularly remarkable in the context of the previously documented structures. The other neck proteins also show detectable, albeit not particularly high (DALI Z-score of around eight for the top hits), structural similarity with analogous previously characterized bacteriophage proteins. The structure of the Nocturne116 HTCP is most similar to what has been observed in neck proteins of the crAss phage and certain podoviruses (42–44), which is surprising considering the substantial evolutionary distance of these viruses. However, the small size and simple four-helix architecture of the HTCP leave a high probability that the similar structures might have arisen independently of each other and do not represent a meaningful evolutionary connection between these viruses. In addition, the Nocturne116 HTCP does not possess extra domains or terminal extensions like its structural similarity hits, and the structure of the assembled HTCP ring is also notably different from the other phages. When compared to the previously determined structures, the HTJP is most similar to a neck protein from *Staphylococcus* phage 80α (24), while the TrP, interestingly, to a type II secretion system protein (45), followed by a tail terminator protein from *Mycolicibacterium* phage Mycofy1 (35). Despite the divergent sequences, the TrP and HTJP appear to be rather typical representatives of their kind and perhaps are only remarkable by their small size, which might bring them close to the functional minimum for such proteins.

At the neck region, the genomic terminus protruding from the head encounters the tail tape measure protein, which fills the lumen of the tail tube; however, there is little conservation in how and where exactly this interaction occurs. The *Bacillus* phage SPP1 employs a “stopper” protein, a structural analog to the HTJP of Nocturne116, which has channel interior-facing loops that act like a diaphragm to control DNA retention or release from the head (46). In *Escherichia* phage T5, the TMP encounters DNA just below the portal, inside the adaptor protein ring (47), while in a *Rhodococcus* phage-like gene transfer agent, the DNA density continues to the tail terminator protein ring (48). In yet other phages, the DNA extends deeper into the tail (49, 50). The cryo-EM reconstructions of the Nocturne116 neck region suggest that in this phage, the DNA terminus extends until the second TTP ring, at which point the single-stranded cohesive (*cos*) overhang of the genome likely asymmetrically interacts with the N-terminus of the TMP. The density features observed inside the Nocturne116 portal, to our knowledge, are dissimilar to the previously determined bacteriophage structures, but despite numerous attempts, we were unfortunately not able to resolve an asymmetric structure of this region to reveal further details. Still, it would seem reasonable to assume that the donut-shaped object in the portal constriction corresponds to averaged-out DNA backbone, which makes an interaction with the portal at that point, while on both sides of the feature, the DNA double helix is in some way disrupted, which manifests in the observed breaks in the density. Of possible importance, a recent cryo-EM study of *Staphylococcus* phage 812 uncovered a transition from B-form DNA to A-form DNA inside the adaptor protein ring (50). While the DNA density inside the 812 neck does not too closely resemble what we observed in our reconstructions, interestingly, the Nocturne116 portal bears certain similarities with the 812 adaptor, which both feature a hydrophobic constriction lined with tyrosine and methionine residues and negatively charged chambers on both sides (Fig. S6). In phage 812, the adaptor is implicated in regulating DNA translocation through the neck, and it cannot be excluded that in Nocturne116, and possibly other c2-like phages, instead the portal serves an analogous role in DNA retention and release.

The Nocturne116 tail tube protein shows a high degree of structural similarity (DALI Z-score ≥ 10) with TTPs from *Pseudomonas* phage JBD30 (51), the flagellotropic phage Chi (52), and mycobacteriophage Douge (53). The core structure of these proteins is nearly identical, and in the tail-assembled state, they use a similar mechanism for extending the outer β-sheet with either a β-hairpin or a single β-strand from the adjacent monomer. A somewhat unique feature of the Nocturne116 TTP appears to be the small C-terminal “decoration” domain sitting on top of the outer β-sheet, although the Douge TTP contains a long loop between the first two β-strands that occupies roughly the same position. Multiple sequence alignment and Alphafold predictions suggest that, besides small insertions or deletions in protein loops, the TTP structure of the other c2-like phages is very similar; however, regarding the tail structure, it can be noted that while the TMPs of the Nocturne116 and Q54 phages are nearly identical in size, the GE1 TMP is 74 residues longer while the c2 protein is 59 residues shorter, suggesting that the number of TTP rings in their tails differs by one or two compared to Nocturne116.

The tail tip, which functions to recognize the host bacterium and initiate the infection, is the most diverged part of the tailed phage virions, often with notable differences between otherwise closely related viruses. Of the structurally investigated *Lactococcus* phages, the tail tip of the skunavirus p2 is the least complex and consists of a distal tail protein that assembles into a hexameric ring at the end of the tail tube, a trimeric hub protein that closes the tube, and six receptor-binding protein (RBP) trimers which are attached to arm-like extensions of the distal tail protein (12). The tail tip structures of other lactococcal phages can be notably more complex, with additional baseplate proteins and a considerable number—up to 54 in phage Tuc2009 (54)—of RBP subunits, resulting in elaborate, megadalton-sized assemblies. In stark contrast, Nocturne116 has the simplest known tail tip structure of any *Lactococcus* virus, composed of only three relatively small components with a total molecular mass of less than 240 kDa. Somewhat atypically, the Nocturne116 phage has two distal tail proteins which assemble into a hetero-hexameric ring with three copies of each; while some phages do encode several DTPs, these usually form two separate rings instead of a single mixed one (47, 55). Both DTPs have a recognizable TTP-like fold but are rather diverged and interestingly, both the DTP1 and the NTD of the DTP2 seem to be structurally more similar with certain phage-like elements (e.g., proteins from a *Photorhabdus* virulence cassette [56], type VI secretion systems [57], or other contractile protein delivery systems [58]) than with phage tail proteins. The CTD of the DTP2 does not show meaningful similarity with previously determined structures, although its overall fold resembles the core of the galectin-like domain in the DTP of the p2 phage. The Nocturne116 baseplate hub protein shows the highest similarity with the gp27 protein from *Escherichia* phage T4 (59) and the hub protein ORF16 from phage p2 (12), although weaker similarity to the contractile injection systems is again detected.

Unlike other components of the virion, the tail tip proteins of the c2-like phages exhibit great variation, which suggests that the organization of the tip structure and, possibly, the host recognition mechanism of these phages might be different. Still, a module of three ORFs encoding a BHP and two DTPs appears to be present in all of these viruses, which suggests that the core structure of their tail tips is nevertheless conserved. The Nocturne116 tail tip proteins share around 50% sequence identity with the questintvirus Q54, its closest known relative, with no notable insertions or deletions, indicating that the tail tip structures of the two phages likely are very similar. In contrast, the respective proteins from ceduoviruses and chertseyvirus GE1 show no detectable sequence similarity with Nocturne116 or Q54 and contain additional domains, suggestive of much larger structural divergence which prevents useful predictions about their organization based on the Nocturne116 structure.

A notable omission from the Nocturne116 tail tip complex is any tail fiber proteins or RBPs, which are typical components of phage particles. We cannot completely rule out the possibility that such proteins might have initially been present in the virion but were then lost during phage purification; however, no candidate proteins resembling RBPs or tail fibers could be located anywhere in the viral genome, even with sensitive structure-based similarity searches. The minimalist tail tip of Nocturne116 thus raises an intriguing question of how, then, the virus manages to bind to its host and deliver the genome into the bacterial cell. The majority of the studied *Lactococcus* siphoviruses use cell wall polysaccharides both for the initial reversible binding step and for a subsequent irreversible interaction that triggers the genome ejection. The ceduoviruses, too, utilize cell wall polysaccharides for initial binding but require a protein receptor for the second phase. The c2 bacteriophage uses a membrane protein Pip as the secondary receptor (60) but, notably, bacteriophage bIL67 from the same genus uses a different, albeit related, protein YjaE for the purpose (61). Receptors of the other c2-like phages, including Nocturne116, are unknown, although it would not be unreasonable to assume that these are also similar kinds of proteins. While this has not been determined experimentally, it is very likely that the strain specificity of the Nocturne116 and Q54 phages differs, which would manifest in sequence variability in tail tip protein regions involved in host recognition. When these proteins of Nocturne116 and Q54 phages are aligned, higher sequence variability maps to a peripheral region of the CTD of the DTP2, a nearby patch on the BHP HD IIIb, and around a pocket at the inter-subunit HD II–HD IV interface (Fig. S7). In this respect, we note that the unaccounted density in the tail tip reconstruction (Fig. 5E) corresponds to one of these variable regions, the HD II–HD IV pockets. While not presently backed by experimental evidence, we find it reasonable to speculate that this density might correspond to a polysaccharide component of the bacterial cell wall which, without triggering the genome release, has remained attached to the tail tip and co-purified with the phage. If correct, such interpretation could imply that the BHP pockets serve as the initial reversible binding sites that allow the phage to stick to the bacterium, and the galectin-like DTP2 CTDs might then serve as the potential irreversible receptor-binding sites. We note that in the closed conformation of the tail tip, as observed in the reconstruction, the HD IIIb of the BHP is positioned like a wedge between HD IV on one side and the DTP2 galectin-like domain on the other (Fig. S7). Conformational changes induced by binding of the galectin-like domain to the protein receptor therefore have the potential to induce a cascade of events during which the HD IIIb reorients and allows the HD IV to move upwards, thus opening the tail tip and releasing the TMP/DNA complex. It remains enigmatic how the DNA would then enter the cell, and further dedicated studies will be required to test this model and arrive at a mechanistic understanding of the infection process of this interesting group of viruses.

## MATERIALS AND METHODS

### Propagation and purification of the bacteriophage

*Lactococcus lactis* LNT bacteria were grown in LB medium at 25°C with slow agitation until the optical density of the culture at 540 nm reached approximately 0.2. Then, Nocturne116 phage was added at a multiplicity of infection of ~1.0, and cultivation was continued for approximately 2 h, at which point bacterial lysis was observed. The resulting lysates were centrifuged at 5,000 × *g* to remove cellular debris, and bacteriophage in the supernatant was sedimented by ultracentrifugation for 2 h in a Type 70 Ti rotor (Beckman Coulter) at 25,000 rpm (64,400 × *g*). The pellets were resuspended in a small volume of SM buffer (20 mM Tris-HCl pH 7.5, 100 mM NaCl, and 10 mM MgSO_4_), and the phage concentrate was layered on top of a preformed five-step gradient of CsCl solutions with densities of 1.7, 1.6, 1.5, 1.4, and 1.3 g/cm^3^ in SM buffer and centrifuged for 20 h at 24,000 rpm (102,574 × *g*) in a SW 40 Ti rotor (Beckman Coulter). The phage-containing band was collected, and excess salt was removed using Amicon Ultra 100 kDa MWCO centrifugal ultrafiltration devices (Millipore). The concentrated samples had a UV absorbance of approximately 10 units at 260 nm and were stored at 4°C until cryo-EM sample preparation.

### Cryo-EM sample preparation and data collection

Three microliters of the purified Nocturne116 bacteriophage was applied to glow-discharged Quantifoil R 1.2/1.3 Gold 300 mesh grids with two additional graphene support layers (total thickness 0.7 nm), blotted for 6 s, and vitrified in liquid ethane using a Vitrobot Mark IV vitrification device. From two suitable grids, a total of 15,554 40-frame movies (dose per frame: 1 e/Å^2^) were collected on a Talos Arctica cryo-electron microscope, equipped with a Gatan K2 Summit direct electron detector and an energy filter with a slit width of 20 eV. The data were collected at a nominal magnification of 165,000×, corresponding to a calibrated pixel size of 0.783 Å, at an accelerating voltage of 200 kV and with the defocus range set to −1.2 to −3.0 μm. Sample vitrification and data collection were performed at CEITEC Masaryk University (Brno, Czech Republic).

### Cryo-EM reconstructions of phage components

Cryo-EM data processing was done using the Relion software package. Initial processing steps (motion correction, contrast transfer function (CTF) estimation, particle picking, and first classifications) were performed using version 4.0.1 of the software (62), while for the latter stages, mostly version 5.0.0 was used. The movies were motion-corrected using Relion’s implementation, followed by CTF estimation of the micrographs with CTFFIND (version 4.1.14) (63) after which 15,349 micrographs with an estimated resolution better than 7 Å and a defocusU value smaller than 35,000 Å were selected. This set of micrographs was used for all subsequent particle extractions.

#### Capsid

From 126 randomly selected micrographs, 270 phage heads were manually picked, subjected to 2D classification, and the class averages were used for automatically picking 47,015 particles from all micrographs. The particles were extracted with a pixel size of 3.132 Å, and after semi-automatic removal of bad particles and two rounds of 2D classification, a set of 33,727 particles was selected and re-extracted with a pixel size of 1.566 Å. The initial model of the capsid was generated using Relion 3.1 (64) with the SGD algorithm, as Relion 4 failed to produce a convincing model with its VDAM implementation. The *de novo* model together with a mask covering the capsid was used as the reference for a 3D refinement with applied C5 symmetry. After refinement of CTF parameters (anisotropic magnification, beam tilt, trefoil, per-micrograph astigmatism, and per-particle defocus), another round of 3D refinement was performed using only local searches starting from a sampling rate of 0.9 degrees; such a refinement strategy was also used for all subsequent 3D refinements. The resolution of the map reached the Nyquist limit at this point; therefore, the particles were re-extracted with the original pixel size of 0.783 Å, and the 3D and CTF refinements were repeated, which at this stage also included refinement of the fourth-order aberrations. Additionally, Ewald sphere correction was applied, which improved the resolution by 0.28 Å. The particles were then subjected to per-particle motion correction (Bayesian polishing), and another round of 3D and CTF refinements. We then performed 3D classification without image alignment, which resulted in over 88% of particles being assigned to a single high-resolution class. The final round of refinement was performed using the 29,796 particles from this 3D class, and additionally, Blush regularization (65) was enabled to further try to improve the resolution. After the final CTF refinement and Ewald sphere correction, the resolution of the structure (FSC_0.143_ criterion) reached 2.63 Å.

#### Portal and neck

Initially, by following a previously described procedure (37), the position of the portal was marked in the capsid reconstruction, and 256-px portal subparticles with subtracted capsid signal were prepared, which allowed reconstruction of a 3.34-Å 12-fold-symmetrized map. To determine the structure of the remainder of the neck region, another vector pointing 90 Å from the portal center toward the tail was defined, and 29,558 448-px subparticles centered at that point were extracted from the micrographs using a modified version of the Localized Reconstruction tool (version 1.4, https://github.com/fuzikt/localrec), which is based on software originally developed by Ilca et al. (66); this tool was also used for all other subparticle extractions during the study. As some of the new coordinates landed very close to the edges of the micrograph, only particles with coordinates at least 398 px from the edges (i.e., not missing more than 50 px from any side) were kept for further processing, which reduced the particle count to 27,997. Using the refined orientations and offsets of the portal subparticles, a 12-Å map was reconstructed and used as the reference for a 3D classification without image alignment, during which the initial C12 symmetry was relaxed to C6. One of the major 3D classes revealed clear densities of the other neck proteins and was used as the reference for 3D refinement, which achieved a resolution of 2.87 Å. During the refinements, Blush regularization was enabled, and a mask covering the entire neck region (including the portal and the first ring of the tail tube) was used. Subsequent per-particle motion correction and further rounds of CTF and 3D refinements allowed the final resolution to increase to 2.83 Å.

The portal structure was refined to a higher resolution together with the other neck proteins than in the previous structure when it was reconstructed alone, and to try to further improve the resolution, a mask covering only the portal and HTCP ring was prepared, and the substructure was re-refined with C12 symmetry enabled. The resulting map offered better-defined density of the peripheral parts of the portal and a modestly improved overall resolution of 2.78 Å.

To obtain the asymmetric structure of the portal-capsid complex, a vector pointing 90 Å back from the central part of the neck toward the head was used to define the coordinates for extracting a set of 360-px subparticles centered at the portal. Using angles and offsets corresponding to the refined C12 structure, a 12-Å map was reconstructed that was further rotationally smoothened by applying 30-fold symmetry, segmented with Segger (67) in Chimera (68), and the region corresponding to the capsid and portal was used to define a mask for subtracting signal from other components, as before. The signal-subtracted particle set was then expanded to C1 with relion_particle_symmetry_expand and subjected to 3D classification without image alignment with a 20 Å low-pass-filtered C12 map as the initial reference. Several of the resulting 3D classes revealed interpretable features of the capsid, and one such class was used as the reference for 3D refinement, initially using all of the signal-subtracted particles, which resulted in a 3.66-Å structure. Subsequent 3D classification without image alignment showed that approximately 74% of the particles correspond to the high-resolution asymmetric structure; these particles were selected and used for another round of 3D refinement. The final refinement round was performed with the original instead of signal-subtracted particles, which resulted in a 3.52-Å map.

Considerable effort was devoted to try to resolve the features inside the portal channel, which involved the use of masks covering different parts of the lumen, symmetry expansion of the various symmetrized neck or portal structures followed by 3D classifications, symmetry relaxation during classification or refinement, and signal subtraction of the neck proteins or portions of the internal density, but unfortunately, none of these approaches were fruitful.

#### Tail tube

Start and end coordinates of 500 tail sections were manually picked from 95 micrographs, and 4,833 tail segments were extracted by assuming an axial rise of 42 Å. The picked particles were used for training and subsequent automated particle picking from all micrographs through the Relion-integrated version of Topaz (69), after which a total of 1,544,037 tail segments were extracted with a pixel size of 2.349 Å. The extracted particles were 2D-classified, and classes containing straight tail tube segments with observable high-resolution features were selected. Particles were checked for duplicates, and those within a distance of less than 20 Å from another particle were discarded, which resulted in a set of 481,849 particles used for the first 3D refinement.

The initial helical parameters of the tail tube (axial symmetry: C6, twist: 17.2°, rise: 42.8 Å) were estimated from the two head–proximal tail rings which were visible in the reconstruction of the neck, and a simulated helical lattice with such parameters was created using relion_helix_toolbox to serve as the initial reference for the 3D reconstruction. After 3D refinement, another 2D classification was performed, and 249,082 particles from classes with straight, high-resolution tail segments were re-extracted with the original pixel size of 0.783 Å. Further processing involved the introduction of a mask covering the central five tail rings of the map, two rounds of 3D and CTF refinements, a Bayesian polishing step, and final 3D and CTF refinements, which resulted in the final map with a resolution of 3.42 Å.

For reconstruction of the head–proximal tail tube segment, two 2D classes centered close to the head–tail connector were selected, which, after deduplication, resulted in a set of 26,465 particles. These particles were subjected to 3D classification using the previously reconstructed tail tube segment as the reference and with C3 symmetry applied, followed by 3D refinement with the best 3D class as the reference. The refined orientations and offsets of the particles were used to guide extraction of another set of 420-px particles from micrographs ~65 Å further down the tail. After a consensus 3D refinement using C3 symmetry, a 3D classification without image alignment was performed, and the best class was used as the reference for re-refinement. Finally, another 3D classification was performed, during which the symmetry was relaxed to C1. The class with the most interpretable features (4,539, or 17.2% of the original particles) was re-refined with limited local searches, resulting in a final map with an estimated resolution of 7.31 Å.

#### Tail tip

The 2D classification of tail segments did not produce convincing classes corresponding to the tail tip; therefore, as an enrichment step, a set of 388,404 particles corresponding only to the previously autopicked tail start and end coordinates were re-extracted with a pixel size of 2.349 Å and subjected to another 2D classification. Several classes putatively corresponding to the tail tip were identified, and particles from the two best classes were selected and used for generation of an initial asymmetric tail tip model, which was afterward aligned to a threefold symmetry axis, symmetrized, and low-pass-filtered to 25 Å. The resulting map was used as the reference for 3D classification of the same particle set, during which a class with discernible higher-resolution features emerged. To find additional particles corresponding to the tail tip, particles from several other 2D classes were added to the previous set, and another 3D classification run was performed using the improved map from the previous classification as the reference. Deduplicated particles from the best class were then used for a 3D refinement, which proceeded to the current Nyquist limit of 4.7 Å. Particles were then re-extracted with the original pixel size of 0.783 Å, and the 3D refinement was repeated, which resulted in a 3.53-Å map. To further improve the reconstruction, a 3D classification without image alignment was performed, and particles belonging to the highest-resolution class were used in another 3D refinement that improved the resolution to 3.39 Å. Similar to other reconstructions, the latter stages of the processing involved rounds of CTF refinement, a Bayesian polishing step, and several 3D refinements, which resulted in a final map with a resolution of 3.05 Å.

### Model building and refinement

Initial models of the head, neck, tail tube, and tail tip were built using ModelAngelo (70) through the Relion interface. The models were then examined and rebuilt as necessary in Coot (version 0.98) (71). All models were real-space refined in Phenix (version 1.21.2) (72, 73). The general refinement strategy involved 3–5 cycles of minimization_global, local_grid_search, nqh_flips, and adp refinements with enabled Ramachandran and rotamer restraints. For all symmetrical structures, NCS constraints between symmetry-related molecules were enforced. For refinement of the capsid structure, additional restraints for the Asn-Lys isopeptide bond were used, which were generated using the program AceDRG (74) from the CCP4 software suite (75). All models were validated using MolProbity (76) either through the Phenix-integrated or standalone versions. Reconstruction parameters, refinement statistics, and quality indicators for the final models are detailed in Table S1. Representative density and Fourier shell correlation curves are provided in Fig. S8.

### Structure analysis

Structural comparisons of Nocturne116 proteins with previously determined structures in the Protein Data Bank were performed using the DALI server (77). *In silico* prediction of protein structures was done using AlphaFold (version 2.3.2) (78). Superpositions of protein subunits for analysis of conformational variability were performed using the SSM algorithm in Coot. Electrostatic properties of the structures were calculated with APBS (version 3.4.1) (79). The structures were visualized, and figures prepared using the open-source version of PyMOL (version 3.0, https://github.com/schrodinger/pymol-open-source) and ChimeraX (version 1.3) (80).

### Mass spectrometry

Proteins from purified Nocturne116 virions were separated in a 15% SDS-polyacrylamide gel; the gel was stained with Coomassie Brilliant Blue G-250, and the bands of interest were excised and cut into small pieces. The gel fragments were washed twice with 0.2 M NH_4_HCO_3_ in 50% acetonitrile for 1 h at 30°C and afterward dehydrated with 100% acetonitrile. The proteins were in-gel digested with trypsin for 3 h at 37°C; the obtained tryptic peptides were mixed with the HCCA matrix according to the manufacturer’s recommendations (Bruker Daltonics) and analyzed using a Bruker Autoflex MALDI-TOF mass spectrometer.

## Data Availability

Coordinates and maps produced during the study have been deposited in the Protein Data Bank and Electron Microscopy Data Bank. The structures are available under the following accession codes: capsid (C5): PDB ID: 9SZ9, EMDB ID: EMD-55362; portal-containing vertex or the particle (C1): PDB ID: 9SZA, EMDB ID: EMD-55363; portal and head-to-tail connector (C12): PDB ID: 9SZB, EMDB ID: EMD-55364; neck (C6): PDB ID: 9SZC, EMDB ID: EMD-55365; tail tube (C6 and helical symmetry): PDB ID: 9SZD, EMDB ID: EMD-55366; tail tip (C3): PDB ID: 9SZE, EMDB ID: EMD-55367, head–proximal tail tube segment: EMDB ID: EMD-58047.

## SUPPLEMENTAL MATERIAL

Supplemental material jvi.00795-26-s0001.pdf Fig. S1 to S8; Table S1.

ASM does not own the copyrights to Supplemental Material that may be linked to, or accessed through, an article. The authors have granted ASM a non-exclusive, world-wide license to publish the Supplemental Material files. Please contact the corresponding author directly for reuse.
