# Supplementary material for: Cryo-EM structure of bacteriophage Nocturne116: insights into the architecture of c2-like Lactococcus viruses
Source: J Virol. 2026 Aug 31;100(9):e00795-26. doi: 10.1128/jvi.00795-26 (PMC13595954; doi:10.1128/jvi.00795-26)
Supplement: Supplementary file 1 [file jvi.00795-26-s0001.pdf]

Supplementary material for

# Cryo-EM structure of bacteriophage Nocturne116: insights into the architecture of c2-like *Lactococcus* viruses

Jānis Rūmnieks[1], Andris Dišlers[1], Kaspars Tārs[1,2]

[1] National Institute of Research and Innovation, Riga, Latvia

[2] Faculty of Medicine and Life Sciences, University of Latvia, Riga, Latvia

**Contents:**

Figures S1-S8

Table S1

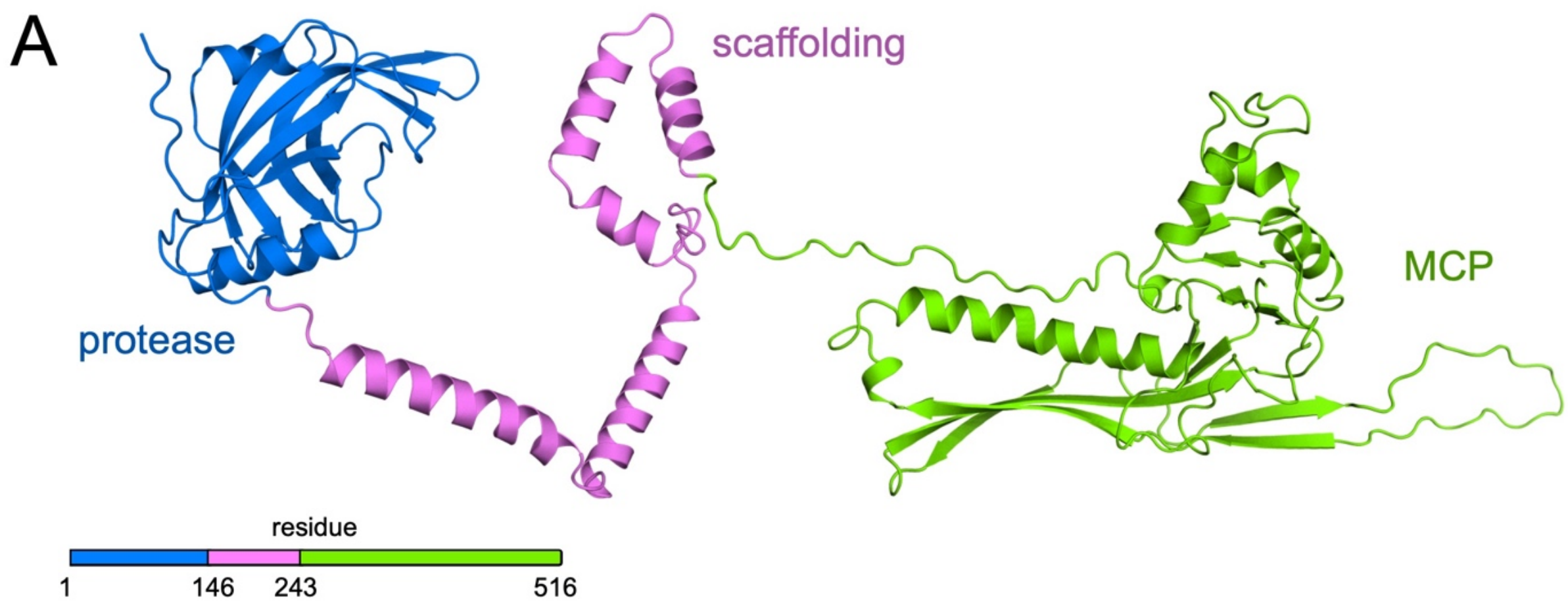


B

```
MCP (ORF18)   243 SITGIPAPTGILKKIHDIVNDRNSILAYIRHENLPTLTVGGDKALSKGEGHTAGTDKNESEIQLETRVLTP-- 313
 VP (ORF19)     1 ----------MTKTIKELF-DKSGISNLITFESSA---------------GMVYIDPNESEYDPETSAFKDLS 47

MCP (ORF18)   314 -QYVYKYIKLPKLVMNKNASEIAGALLTYVLNRLPQMVIFAVNRAIIMGDVTGVSKTQIYPVVGDTWATN-VG 384
 VP (ORF19)    48 VEQFSKVIEIPRQVYQKEG-----QIEL-YKNRLVAEAAAELLKAIIVGGSTDFTSDSVVSLSEDALEVVPSG 114

MCP (ORF18)   385 ---EKETVIDLIETLSVSTPKAYDSSLVISRADWSAVRFT--KDKNGRERFLGLSDAELANHFGFERVLITTA 452
 VP (ORF19)   115 ATPTQATVQDLLL-----KNKALDDSLYINTFTADTLKTAETAAYDLADTFVTIKGAP--TIYGFTI------ 174

MCP (ORF18)   453 VDAGTIVSLDGYVTNGSRGMEFEEGRILVENNKEYLFEMPISGSLEYKEMAAYGTIKKPVTGGE-----     516
 VP (ORF19)   175 -------PDKSYTLY--INEEFETEPQWSNNKLRIRYTLIAQ--------------GMPTATGHAVTAL     220
```

**Figure S1. The ORF18 and ORF19 products of the Nocturne116. A,** Model of the full-length ORF18 product. Structure of the protease and scaffolding domains were predicted using Alphafold and combined with the experimentally determined MCP structure. **B,** Sequence alignment of the ORF18 MCP domain and VP. Residues identical in both proteins are highlighted.

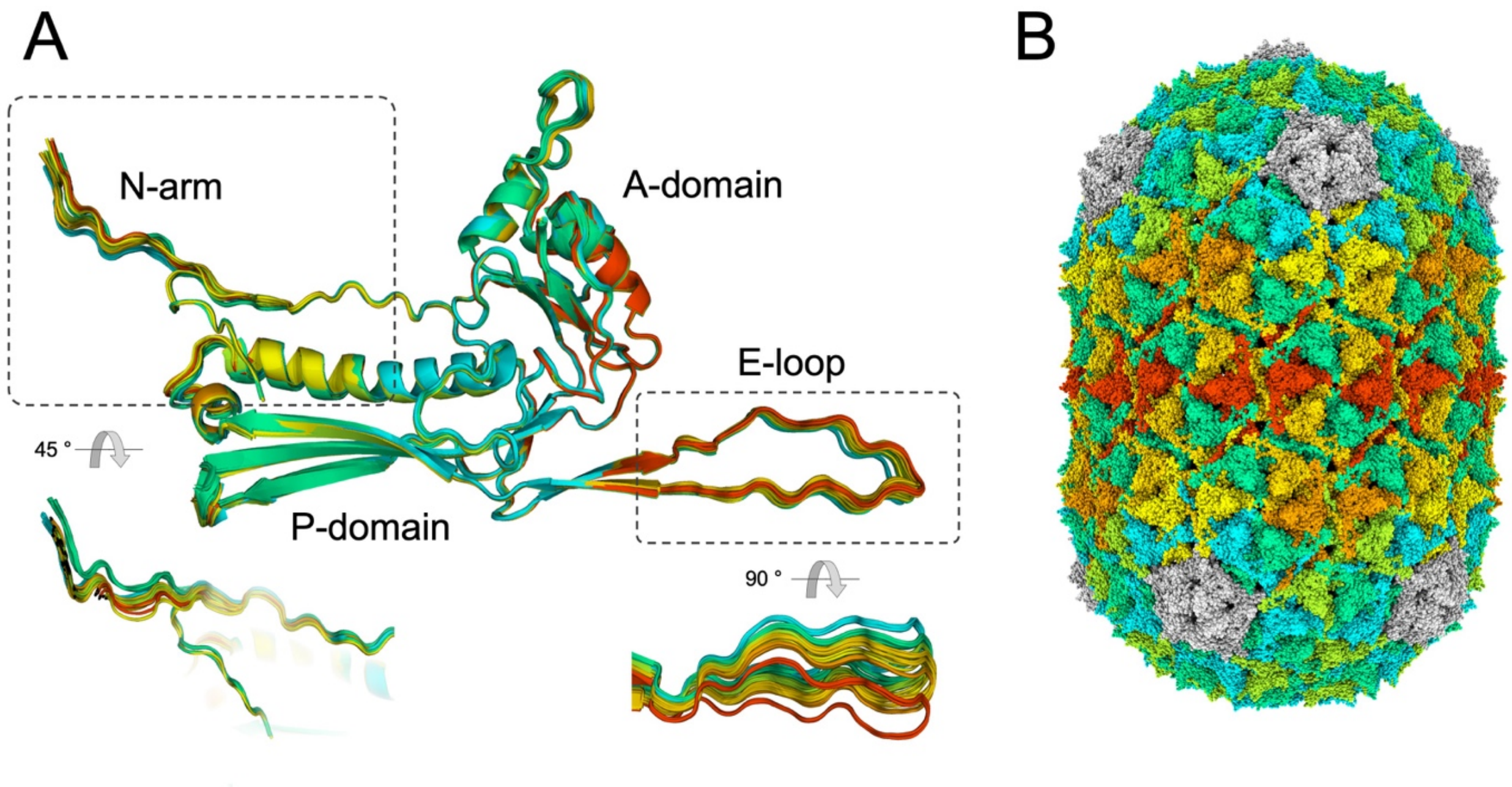


**Figure S2. Conformational variability of the major capsid protein in the Nocturne116 particle. A,** Superposition of MCP monomer structures within the asymmetric unit of the capsid. The two regions with the largest observed conformation variation are indicated. **B**, Distribution of the different MCP conformations within the capsid. The MCP monomers in both panels are rainbow-colored according to the relative angle of the E-loop with respect to the P-domain.

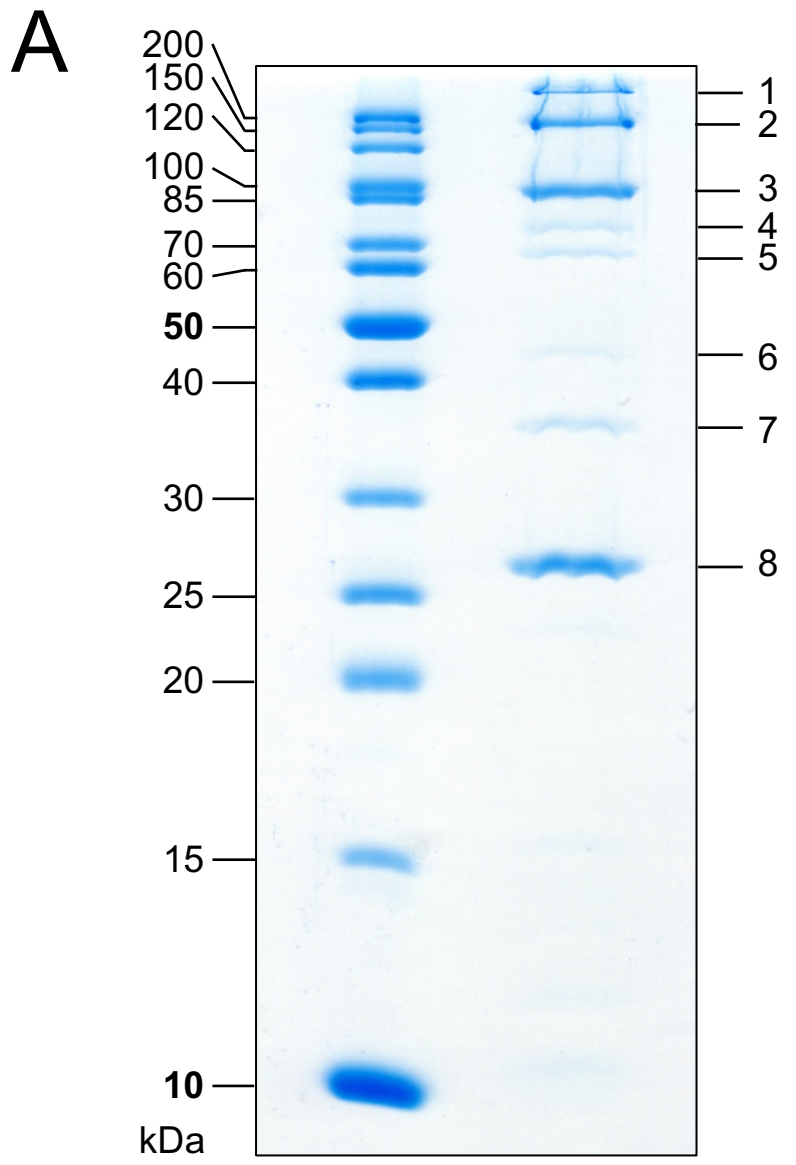


B

| band | peptide mass fingerprint analysis | | | molecular weight (kDa) | |
|---|---|---|---|---|---|
| | identified protein | | detected peptides | theoretical | observed |
| 1 | ORF18 | MCP | 6 | 30 | >200 |
| 2 | ORF18 | MCP | 10 | 30 | 180 |
| | ORF19 | VP | 1 | 24 | 180 |
| 3 | ORF18 | MCP | 9 | 30 | 90 |
| | ORF19 | VP | 1 | 24 | 90 |
| 4 | ORF23 | TMP | 6 | 81 | 75 |
| | ORF18 | MCP | 5 | 30 | 75 |
| 5 | ORF18 | MCP | 4 | 30 | 65 |
| 6 | ORF27 | BHP | 4 | 43 | 45 |
| 7 | ORF17 | PP | 3 | 35 | 35 |
| 8 | ORF20 | TTP | 3 | 24 | 27 |

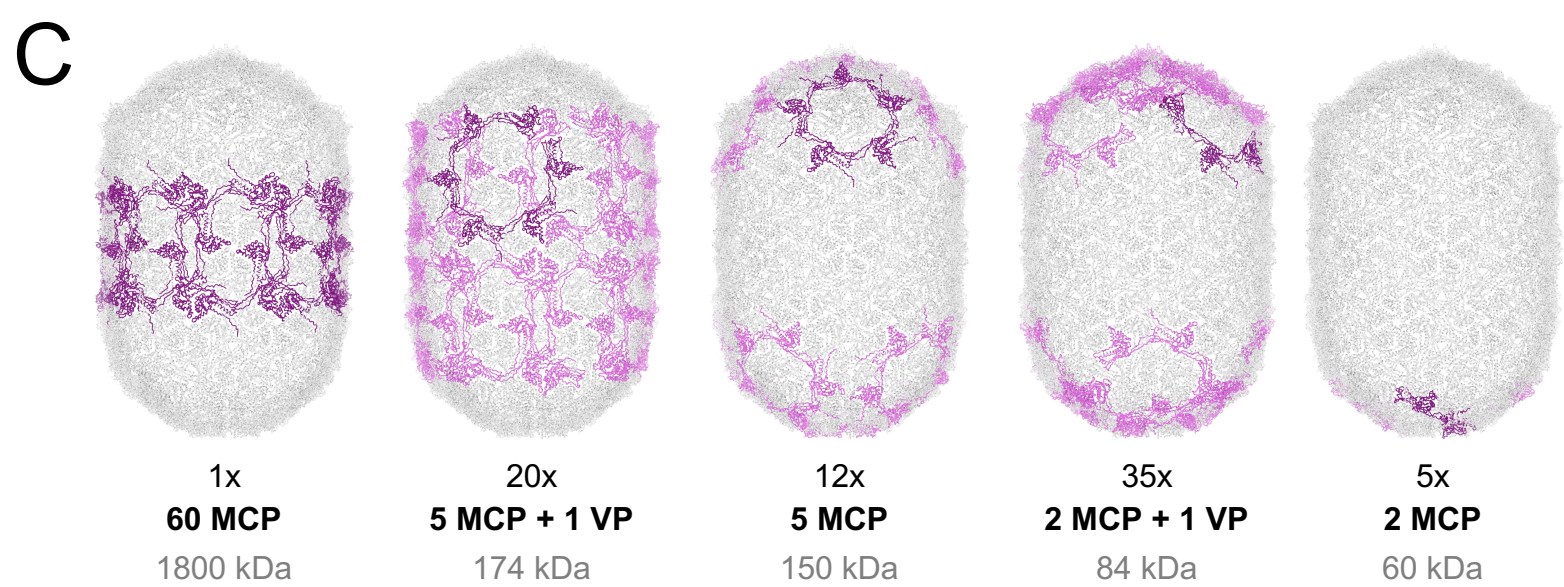


**Figure S3. Covalent crosslinks in the Nocturne116 capsid. A**, SDS-PAGE analysis of purified Nocturne116 virions (right lane). **B**, Mass spectrometry analysis of tryptic peptides from the top eight gel bands. The identified proteins, number of the detected peptides, and the agreement between the predicted and experimentally observed molecular weight of the proteins are detailed. **C,** Covalently linked and catenated capsid protein species in the Nocturne116 particle. The location of each of the oligomeric species in the capsid is highlighted, with the number of such oligomers in the particle, their composition, and approximate theoretical molecular weight provided below. In each case, a single covalently linked oligomer is emphasized by a darker shading; in case of the cylindrical midsection, the entire band of ten catenated rings is considered as a single oligomer.

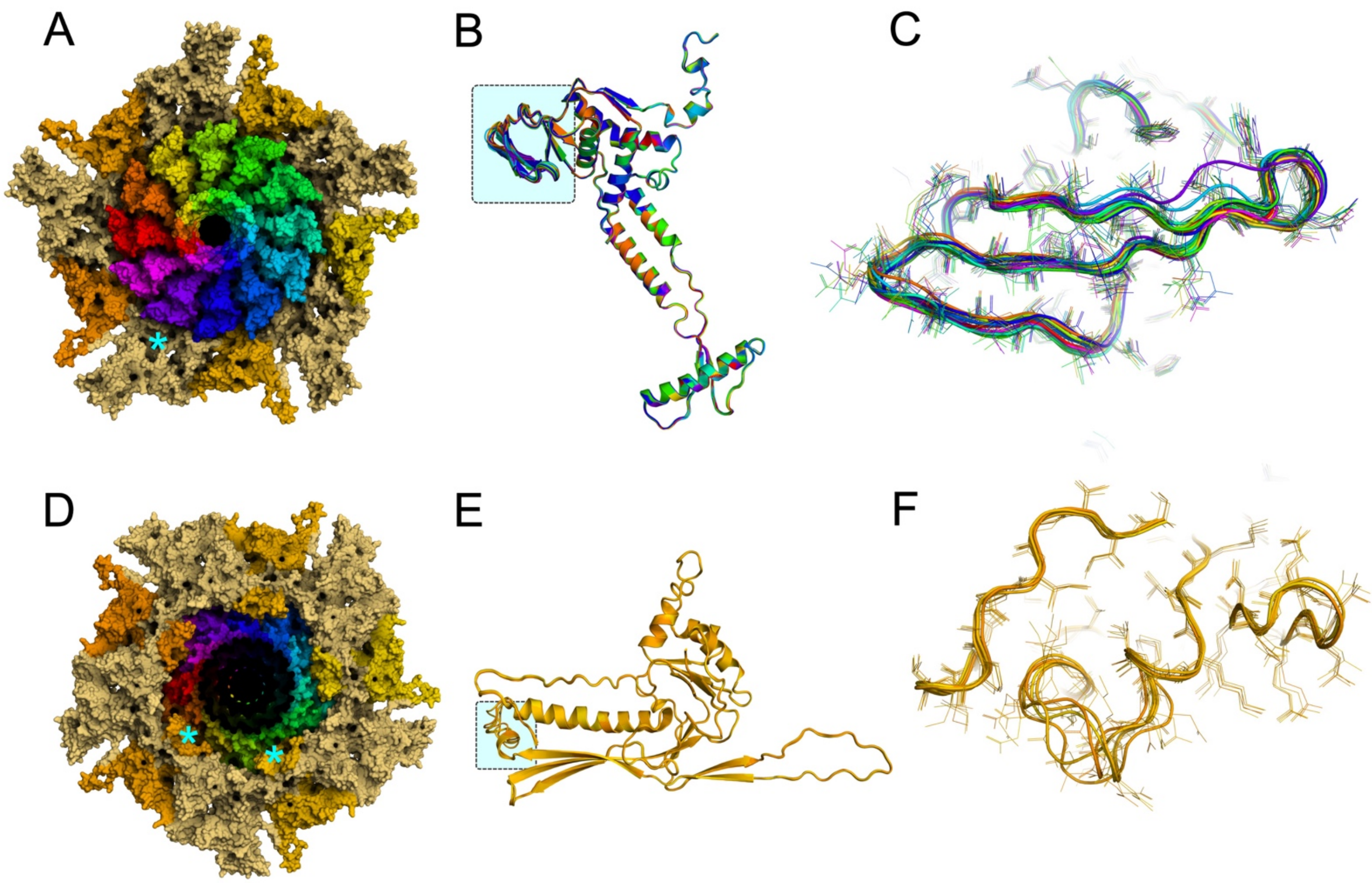


**Figure S4. Asymmetric interaction of the portal and the capsid. A,** Molecular surface representation of the portal vertex, viewed from inside of the capsid. The portal protein (PP) chains are rainbow-colored, and the surrounding major capsid protein (MCP) molecules are shown in shades of orange. The PP subunit which undergoes the largest conformational change upon interacting with the capsid is marked with an asterisk. **B**, Superposition of PP subunits from the asymmetric structure. The region of the wing domain with the largest conformational differences between subunits is highlighted; the subunit colors are consistent with panel A. **C**, A close-up view of the peripheral wing domain region of the superimposed PP subunits. **D**, Molecular surface representation of the capsid-integrated portal as observed from the outside. For an unobstructed view, the clip domains are clipped off. The two MCP subunits which exhibit the biggest conformational change are marked by asterisks. **E,** Superposition of the five portal-surrounding MCP molecules which interact with the base of the portal's wing. Monomer coloring is consistent with panel D. The region of the MCP P-domain which is involved in the interaction is highlighted. **F**, A close-up view of the peripheral P-domain region highlighted in panel E.

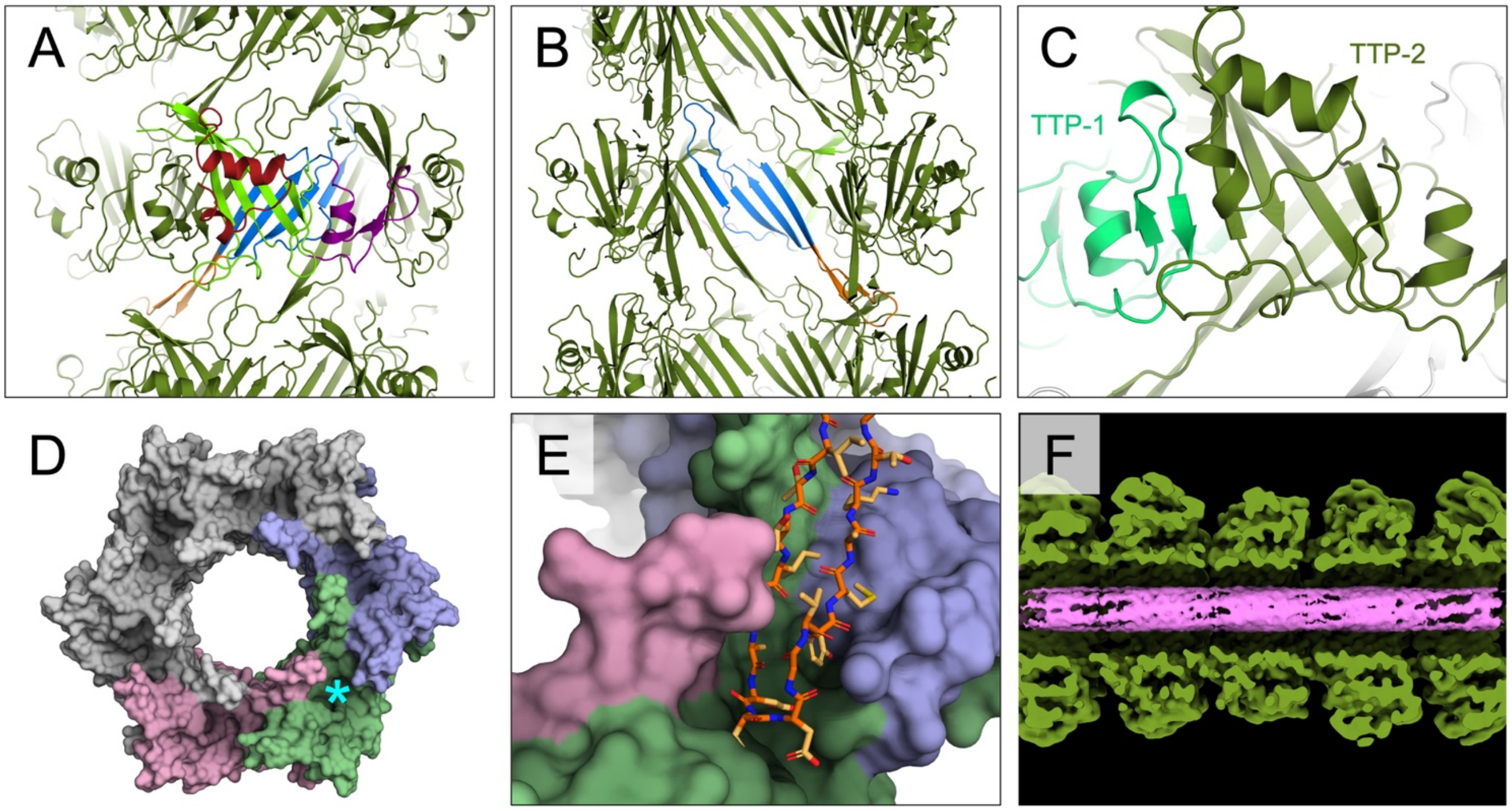


**Figure S5. Structure of the Nocturne116 tail tube. A,** Subunit organization within the tail tube. The tail tube is viewed from the outside, perpendicular to the central tail axis. A tail tube protein (TTP) monomer in the center is colored as in Fig. 5C in the main text. **B**, Structure of the tail lumen. A cut-away view of the tail tube with a single TTP subunit highlighted. **C**, Inter-subunit interactions around the TTP ring. A β-hairpin in the lateral loop of one of the subunits (TTP-1) extends the outer β-sheet of a neighboring monomer (TTP-2) to form a joint six-stranded β-sheet. **D**, The interface between adjacent TTP rings. Three neighboring subunits collectively form a binding pocket for the long loop from the adjacent ring. Three representative subunits are colored pink, light green and light blue, with the pocket marked with an asterisk. **E**, A close-up view of the binding pocket in panel D with the docked long loop shown in orange. **F**, Additional density inside the tail lumen. Reconstruction of the tail tube reveals averaged density of the tail tape measure protein, shown in light magenta. Density of the TTP subunits is colored olive green.

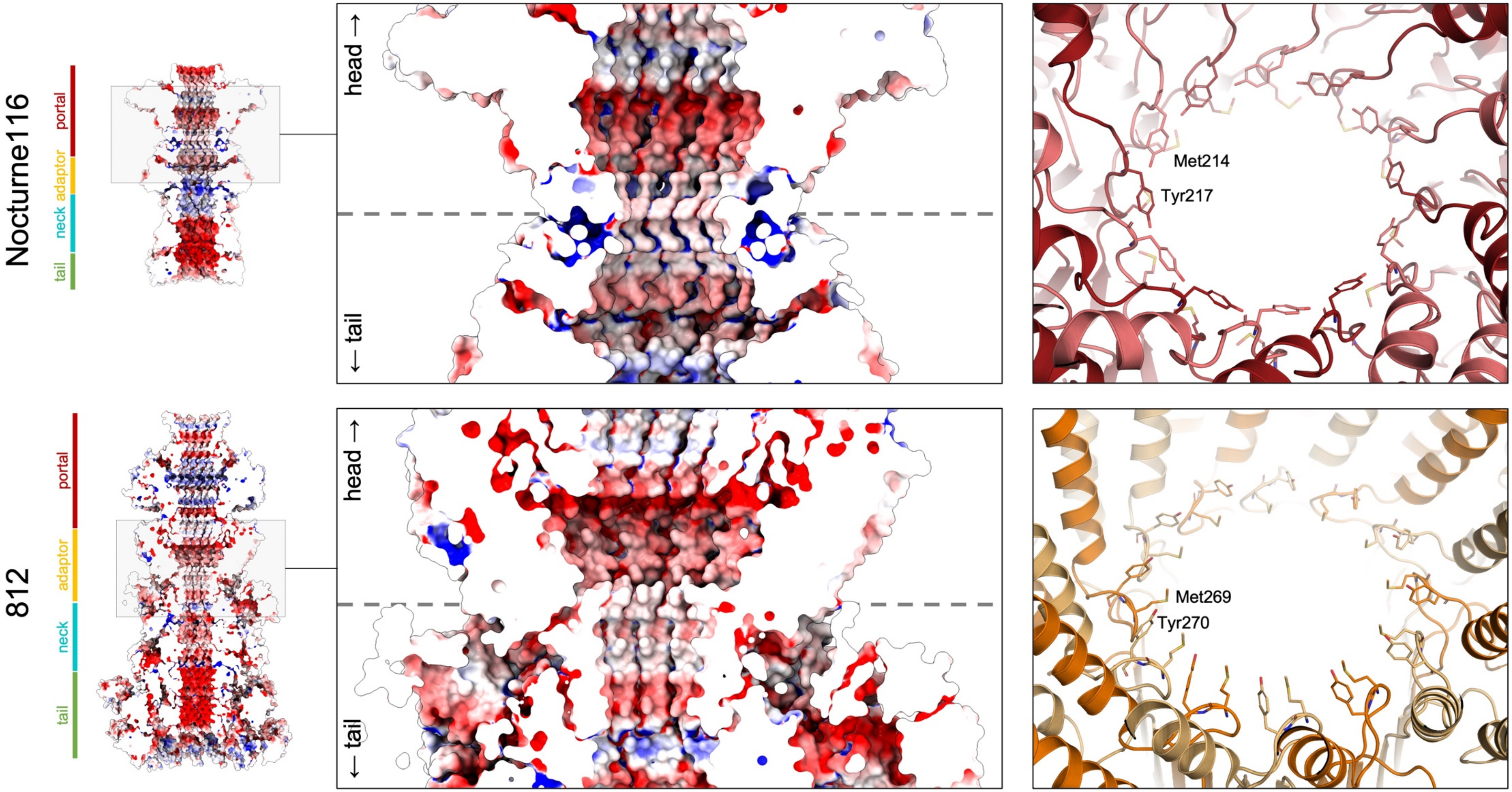


**Figure S6. Similar properties of the internal channel connecting the head and the tail in Nocturne116 and *Staphylococcus* phage 812.** Left, cut-away views of the head-tail connection of the two phages, colored according to their computed electrostatic potential. The approximate boundaries of the head-tail interface regions are indicated on the left. Center, an enlarged view around the constriction inside the portal in Nocturne116 or the adaptor in phage 812, with the narrowest point in the structure indicated by a dashed line. Right, a close-up view of the neck channel around the constriction, with a ring of tyrosine and methionine residues lining the channel emphasized.

**DTP1**

```
Nocturne116     1 MSIKIFKTTPAAMYVISNSENVDIKPEGLELCVAEIDDLIEPSGTFTLTLSENDPFLIQENQLYTLRDEF  70
        Q54     1 MSYKIFKSTTASMYDVSSSSNIDIKPTGLDFIVLSISDHAEPGEDFTITLSENEPLLTYLNQLYTIRDNF  70

Nocturne116    71 GATKWSVNRATHITPFFVSSVRREFGIVKYECTKVYKSFLEMTGLYSGELL                   121
        Q54    71 NENSY------NITPFFVTGSDREFGKITYTCTKVYKGYLIVPGIFEGEIL                   115
```

**DTP2**

```
Nocturne116     1 MKKAKIEGWS--LVDLWVDETPFQSDKSVNIYGFDAFPVLIKSWASQRDRLIITIDEGNGAISLFVAKSK 68
        Q54     1 MKKVTLEAYDSQGMEFFVENTPKQEDKTIDVFGYDTFKMLSKSWASQRDQFIITINEGGAAISLLLVKSK 70

Nocturne116    69 RGNGYVGRKRESGELRVGRIFDIVSPIYYIKNPVRGDGSSPAVKYITTYSKDSDVKACMFFPTAVAELQV 138
        Q54    71 SGNGYVKRELESGTHAVGRIFDIVSPIYYIKNPVRGSAT---LTEMTTYAYNSNIRRCMFFPEPVTKMQM 137

Nocturne116   139 LVNGYTPGDELTKTYGKGFGVAGTSTFYESMSCEFSGEIAGFDSCQIRNTTDSTGATRAAYIGGEYQIL  207
        Q54   138 FFNQYPMNTAHNVPYGRGFGAAGRSFFYESISFEPDLAIQK-DLTIVINNNNTDGATPKTFIGGEYLIL  205
```

**BHP**

```
Nocturne116     1 MTTHSLKLLHLQGRSTPWQNSPQVTVSVIQDRWTFDKGREIEFMAMGLPQSFIFIVDSKIQDQEGQRAGD  70
        Q54     1 MTAHTLKMYHLTSRTLSWRSSPEVIVTVVSDRWQFDTGREIEFYAMNMPDSFAFVVDGLRNNQDGQRWGD  70

Nocturne116    71 CRAGSWCGQATRIGLNRYQGIQASGTNNEASGLIAFGTANSTSFGKVSTSNVSGIALGANIDVALGQTAA 140
        Q54    71 SRSGSWAGIASKIAPNKYQGIQASGTSNEASGLIAYGTAVKVAFGHANGNEAGTLPLGMYSDGALVEGYS 140

Nocturne116   141 ANVSSGAVADRMSFPEFGRPKTPNARIRTLAFGNAVDQATKTIRSGPSVVRRSMYEFGGDFATTQFYPRY 210
        Q54   141 KDVVIGSNTARFTKPEFGNPADPDPTIRTVLFGDAVDQQTKTIRQGASKTREAMYRFSGALATTQFYPRT 210

Nocturne116   211 DSNIAVGGVTYGSCYTADYQGLPMGAIKASALPISEGKVSEKLDGDGRTAVWRVWAIAGAENLTPYKYHY 280
        Q54   211 DSNNAVGGVTFAYGKTVDQVILAELTLMASAAPISDGAVSEKKDGNGRVATWKQYFLAGASNVSPYQPHY 280

Nocturne116   281 LVMYVEADGTLSFGASTGSTPNSTNGDSVFSEAYTIFSDDKFNTANNSENQDILKQAYYAAKTKSQNFTV 350
        Q54   281 LLLDIGENGSFVFGSNSGFTPGPN-GAYLGFAALTIFSDDKQNTADTLENRAILTNLYYSTKARNRSYVI 349

Nocturne116   351 KRPIVVPNAFFPLTEKTQKGHLITQSWIISADFINHEYRIGE                            392
        Q54   350 KRPMVVPHRFNVVSNKTLLGYALSESWLISADFINYEYTIGE                            391
```

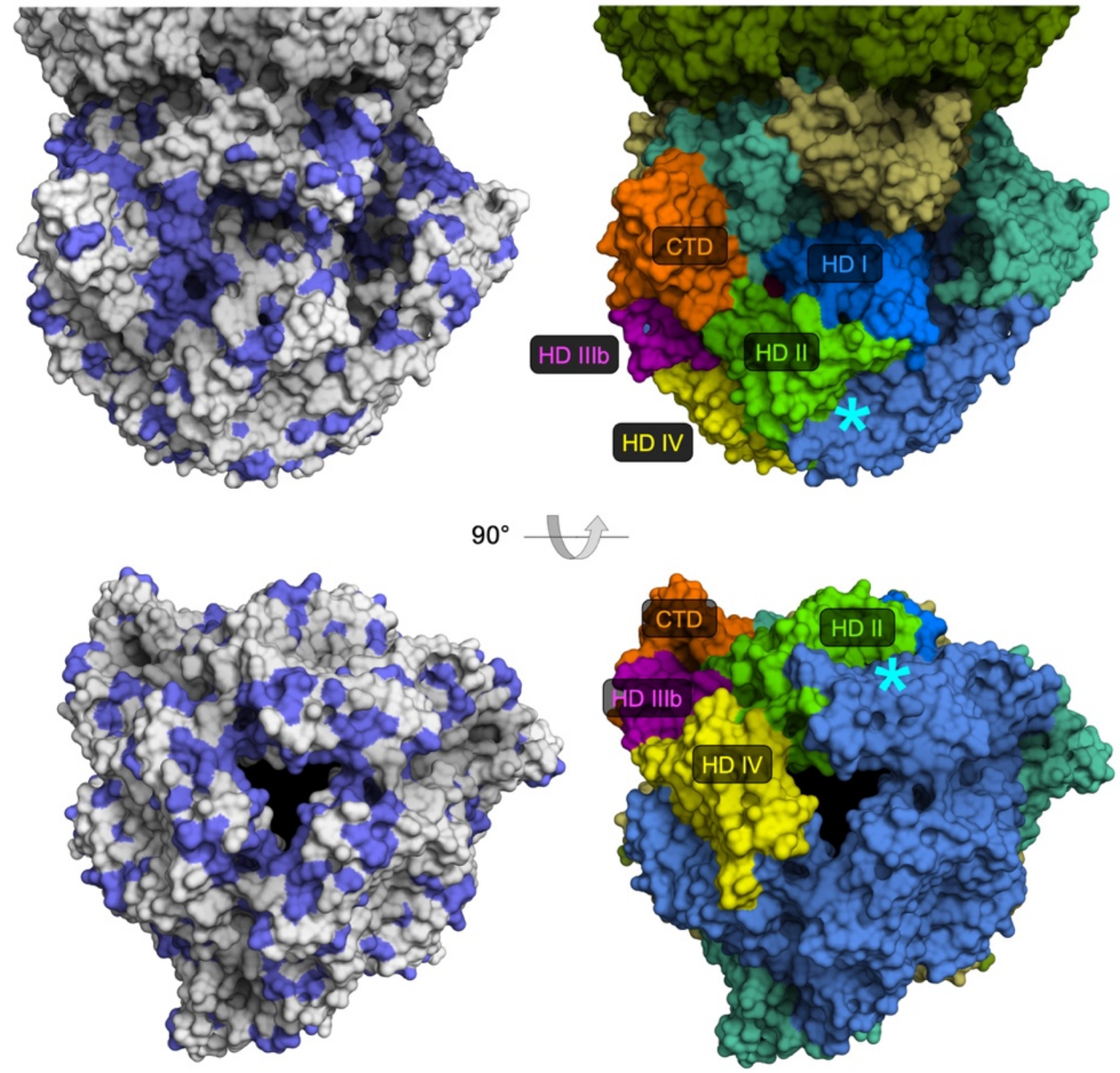


**Figure S7. Conserved regions of the Nocturne116 tail tip.** Left panel, sequence alignment of the three tail tip proteins from Nocturne116 and its closest known relative, *Lactococcus* phage Q54. Identical residues of the proteins are highlighted. Right panel, mapping of the conserved regions onto the Nocturne116 tail tip. The tail tip is shown as viewed from the front and from the bottom. On the left side, residues identical to the Q54 phage are colored consistent with the sequence alignment; on the right side, the tail tip is shown in the same orientation but colored according to the constituent proteins. The distal tail protein 1 (DTP1) in shown in khaki, the distal tail protein 2 (DTP) in aquamarine and the baseplate hub protein (BHP) in blue, except for one of the BHP subunits which is colored according to its domain structure (HD I: bright blue; HD II: green; HD IIIb: dark magenta; HD IV: yellow); in addition, the C-terminal domain (CTD) of an adjacent DTP2 molecule is indicated in orange. The pocket in which the unaccounted density was observed in the tail tip reconstruction (Fig. 5E) is marked with an asterisk.

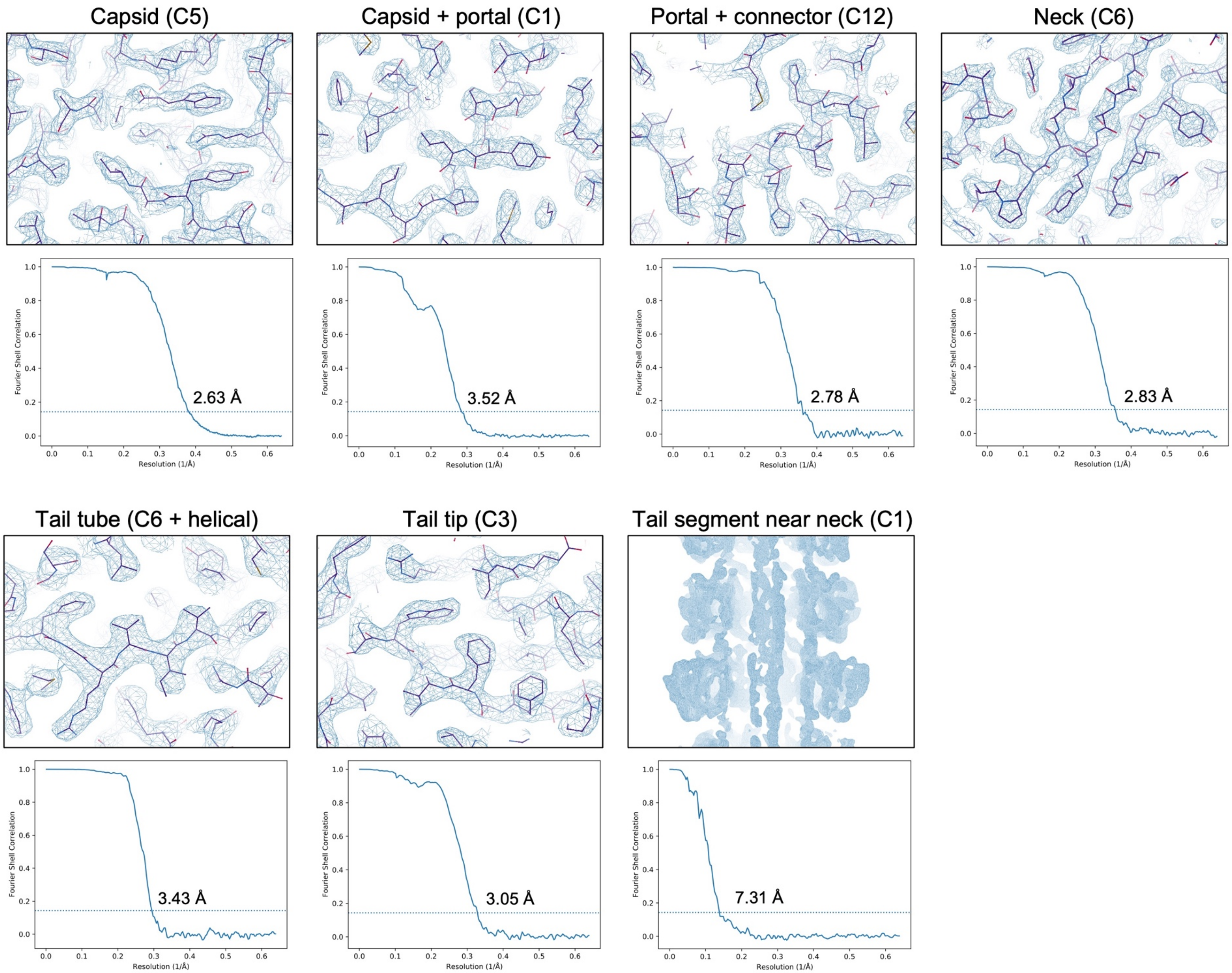


**Figure S8. Representative density and estimated resolution of the Nocturne116 virion subcomponents.** Top, representative density and the fitted model for the high-resolution structures. Bottom, Fourier shell correlation curves as a function of resolution. The 0.143 threshold used for estimating the achieved resolution is indicated as a dotted line.

| | capsid | capsid + portal | portal + connector | neck | tail tube | tail tip | tail segment near neck |
|---|---|---|---|---|---|---|---|
| **Accession numbers** | | | | | | | |
| Coordinates (PDB) | 9SZ9 | 9SZA | 9SZB | 9SZC | 9SZD | 9SZE | |
| Maps (EMDB) | EMD-55362 | EMD-55363 | EMD-55364 | EMD-55365 | EMD-55366 | EMD-55367 | EMD-58047 |
| **Reconstruction** | | | | | | | |
| Symmetry | C5 | C1 | C12 | C6 | C6 + helical | C3 | C1 |
| Number of particles | 29793 | 20447 | 27997 | 27997 | 249082 | 10559 | 4539 |
| Map resolution (FSC 0.143) | 2.63 | 3.52 | 2.78 | 2.83 | 3.43 | 3.05 | 7.31 |
| Voxel size (Å) | 0.783 | 0.783 | 0.783 | 0.783 | 0.783 | 0.783 | 0.783 |
| Box size (px) | 1024 | 360 | 448 | 448 | 420 | 420 | 420 |
| Helical twist (°) | | | | | 16.08 | | |
| Helical rise (Å) | | | | | 42.77 | | |
| **Refinement** | | | | | | | |
| Model composition | | | | | | | |
| Chains | 355 | 39 | 24 | 42 | 18 | 18 | |
| Residues | 92090 | 8208 | 4248 | 6606 | 3780 | 3465 | |
| Atoms | 712345 | 63846 | 33156 | 51582 | 28656 | 26622 | |
| Fit of map and model | | | | | | | |
| CC (masked) | 0.86 | 0.84 | 0.88 | 0.84 | 0.88 | 0.88 | |
| Masked map-model FSC | 2.67 | 3.54 | 2.79 | 2.87 | 3.41 | 3.1 | |
| RMSD from ideal geometry | | | | | | | |
| bond lengths (Å) | 0.002 | 0.002 | 0.001 | 0.001 | 0.002 | 0.001 | |
| bond angles (°) | 0.416 | 0.366 | 0.337 | 0.35 | 0.389 | 0.385 | |
| Average B-factor | | | | | | | |
| protein ($Å^2$) | 28.51 | 64.63 | 43.43 | 44.67 | 40.75 | 53.31 | |
| Ramachandran plot | | | | | | | |
| favored (%) | 98.54 | 98.53 | 99.04 | 98.59 | 98.08 | 98.43 | |
| allowed (%) | 100 | 100 | 100 | 100 | 100 | 100 | |
| Z-score | 0.5 | 1.18 | 1 | 0.85 | -0.17 | -0.05 | |
| Rotamers | | | | | | | |
| favored (%) | 92.85 | 93.18 | 96.44 | 94.35 | 89.88 | 92.73 | |
| outliers (%) | 0.06 | 0.01 | 0 | 0.1 | 0 | 0.11 | |
| Model quality indicators | | | | | | | |
| Clashscore | 2.82 | 3.06 | 3.34 | 3.06 | 4.78 | 3.61 | |
| MolProbity score | 1.07 | 1.1 | 1.12 | 1.1 | 1.25 | 1.15 | |

**Table S1. Reconstruction parameters, refinement statistics, and model quality indicators for the Nocturne116 virion subcomponents.**
